# Exploring Differential Transcriptome between Jejunal and Cecal Tissue of Broiler Chickens

**DOI:** 10.3390/ani9050221

**Published:** 2019-05-07

**Authors:** Micol Bertocchi, Federico Sirri, Orazio Palumbo, Diana Luise, Giuseppe Maiorano, Paolo Bosi, Paolo Trevisi

**Affiliations:** 1 Department of Agricultural and Food Sciences (DISTAL), University of Bologna, 40126 Bologna BO, Italy; micol.bertocchi2@unibo.it (M.B.); federico.sirri@unibo.it (F.S.); diana.luise2@unibo.it (D.L.); paolo.trevisi@unibo.it (P.T.); 2 Department of Agricultural, Environmental and Food Sciences, University of Molise, 86100 Campobasso CB, Italy; maior@unimol.it; 3 Division of Medical Genetics, Fondazione IRCCS Casa Sollievo della Sofferenza, 71013 San Giovanni Rotondo FG, Italy; o.palumbo@operapadrepio.it

**Keywords:** broiler chicken, jejunum, cecum, transcriptome, microarray

## Abstract

**Simple Summary:**

Intestinal main functions and different gut tract peculiarities in poultry are known. However, a wider view at the molecular level in terms of functional genes may contribute to deepening knowledge on less considered biological aspects, with possible differentiation in gene expression and functionality between gut tracts. This experiment aimed to extend the exploration of chicken gut functional aspects by scouting differential gene expression in the jejunum and cecum, which may help in the detection of new interesting functions from a biological point of view. The work identified key aspects linked to gut biological processes that may be worthy of further investigations in experimental studies considering factors which may specifically address peculiarities of the different chicken gut tracts at the molecular level.

**Abstract:**

The study proposed an exploratory functional analysis on differential gene expression of the jejunum and of cecum in chickens. For this study, 150 Ross 308 male chickens were randomly allotted in six pens (25 birds/pen) and fed the same commercial diet. From 19 birds of 42 days of age, jejunum and cecum mucosae were collected for RNA extraction for transcriptome microarray analysis. Differentially expressed genes (DEGs) submitted to DAVID (Database for Annotation, Visualization, and Integrated Discovery) and Gene Set Enrichment Analysis (GSEA) software evidenced enriched gene clusters for biological functions differentiated in the tissues. DAVID analysis in the jejunum showed enriched annotations for cell membrane integral components, PPAR (peroxisome proliferator-activated receptor) signaling pathway, and peroxisome and lipid metabolism, and showed DEGs for gluconeogenesis, not previously reported in chicken jejunum. The cecum showed enriched annotations for disulfide bond category, cysteine and methionine metabolism, glycoprotein category, cell cycle, and extracellular matrix (ECM). GSEA analysis in the jejunum showed peroxisome and PPAR signaling pathway-related gene sets, as found with DAVID, and gene sets for immune regulation, tryptophan and histidine metabolism, and renin–angiotensin system, like in mammals. The cecum showed cell cycle and regulation processes, as well as ECM receptor interaction and focal adhesion-related gene sets. Typical intestinal functions specific for the gut site and interesting functional genes groups emerged, revealing tissue-related key aspects which future studies might take advantage of.

## 1. Introduction

Primary functional specificities of the small and large intestine, which are important for the proper activities of the gut, are considered well known and were explored for several decades. However, the modern molecular approaches can improve the knowledge about the presence and the relative expression of thousands of genes in tissues. Nevertheless, higher attention is given to a limited number of genes and protein products [1,2], due to their frequent presence in previous studies, although this choice of genes does not necessarily reflect their biological relevance. Furthermore, a more in-depth exploration of gut functional aspects may be interesting in order to provide hints for action (e.g., dietary strategies) to favor gut homeostasis [3], given the important role of the intestine in the development of possible metabolic diseases. A careful survey on differential gene expression may help scout new interesting functions and identify potential markers for testing various experimental factors.

For humans, the gene expression in different tissues is currently collected by some portals developed by medical projects, such as GTEX (gtexportal.org, The Broad Institute of MIT and Harvard, Cambridge, MA, USA) or BioGPS (biogps.org, The Scripps Research Institute, La Jolla, CA, USA); differential gene expression between different gut tracts was explored and discussed in mice (from the stomach to the colon [4]) and in pigs (among different gastric mucosae [5], and along the small intestine [6]), while a lack of such a survey was detected in chickens. In addition, the spatial differences in dominant bacteria along the gut, and particularly between the small and large intestine [7] suggest that these variations may affect the expression of genes in the different chicken gut segments. Furthermore, while in other species the cecum is not considered a key intestinal tract, in chicken, it has a key role for the digesta fermentation, and further information on its molecular functionality may be important.

The aim of the present research was, thus, to evidence the differential tissue gene expression of the jejunum and of the cecum of chickens at 42 days of age.

## 2. Materials and Methods

### 2.1. Animals and Sampling

The experiment design was approved by the Ethical Committee of the University of Bologna on 3 May 2017 (ID363/2017-PR).

A total of 150 Ross 308 male chicks were reared at the experimental facility of the University of Bologna within an environmentally controlled poultry house, and they were randomly allotted in six pens of 6 m^2^ each (25 birds/pen). All the chicks were vaccinated against coccidiosis, infectious bronchitis virus, Marek’s disease virus, and Newcastle and Gumboro disease. Stocking density was defined according to the European legislation in force (European Commission, 2007) to simulate the environmental conditions usually adopted in the intensive production system. Each pen was equipped with two circular pan feeders able to guarantee at least 2 cm of front space/bird and 10 nipples, while the floor was covered with chopped straw (2 kg/m^2^). According to the legislation in force (European Commission, 2007), birds received a 23-h light/1-h dark cycle with artificial light from zero to seven days and in the last three days before slaughter, whereas an 18-h light/6-h dark photoperiod was adopted in the remaining days.

All the birds received the same standard commercial diet composed of three feeding phases: starter (0–10 days), grower (11–25 days), and finisher (26–42 days) (Table S1, Supplementary Materials).

At slaughter age (day 42), 24 birds (four birds/replicate) were selected for body weight homogeneity. The jejunum and cecum mucosae were collected from selected birds by gently scraping tissues after rinsing in PBS (phosphate buffered saline) to remove residues of digesta. Mucosa samples were immediately frozen in liquid nitrogen and then stored at −80 °C.

### 2.2. RNA Analysis

Total RNA from each individual sample was extracted using a GeneJET RNA Purification Kit (Thermo Scientific, Waltham, MA, USA) according to the manufacturer’s instructions. RNA quantity and quality were evaluated using a Nanodrop ND 1000 spectrophotometer (Nanodrop Technologies Inc., Wilmington, DE, USA) and agarose gel electrophoresis, respectively. The RNA integrity was evaluated through an Agilent Bioanalyzer 2100 (Agilent Technologies, Santa Clara, CA, USA). Samples out of five subjects were discarded since RNA integrity was compromised in one of the two tissues. Thus, the whole-transcriptome microarray analysis was then performed individually on a total of 38 samples considering both tissues (i.e., 19 subjects per tissue), using an Affimetrix© GeneChip Chicken Gene 1.0 ST Array, and hybridized arrays were scanned on an Affimetrix© GeneChip Scanner 3000 7G System (Affymetrix, Santa Clara, CA, USA).

### 2.3. Data and Statistical Analysis

**Figure 1:**
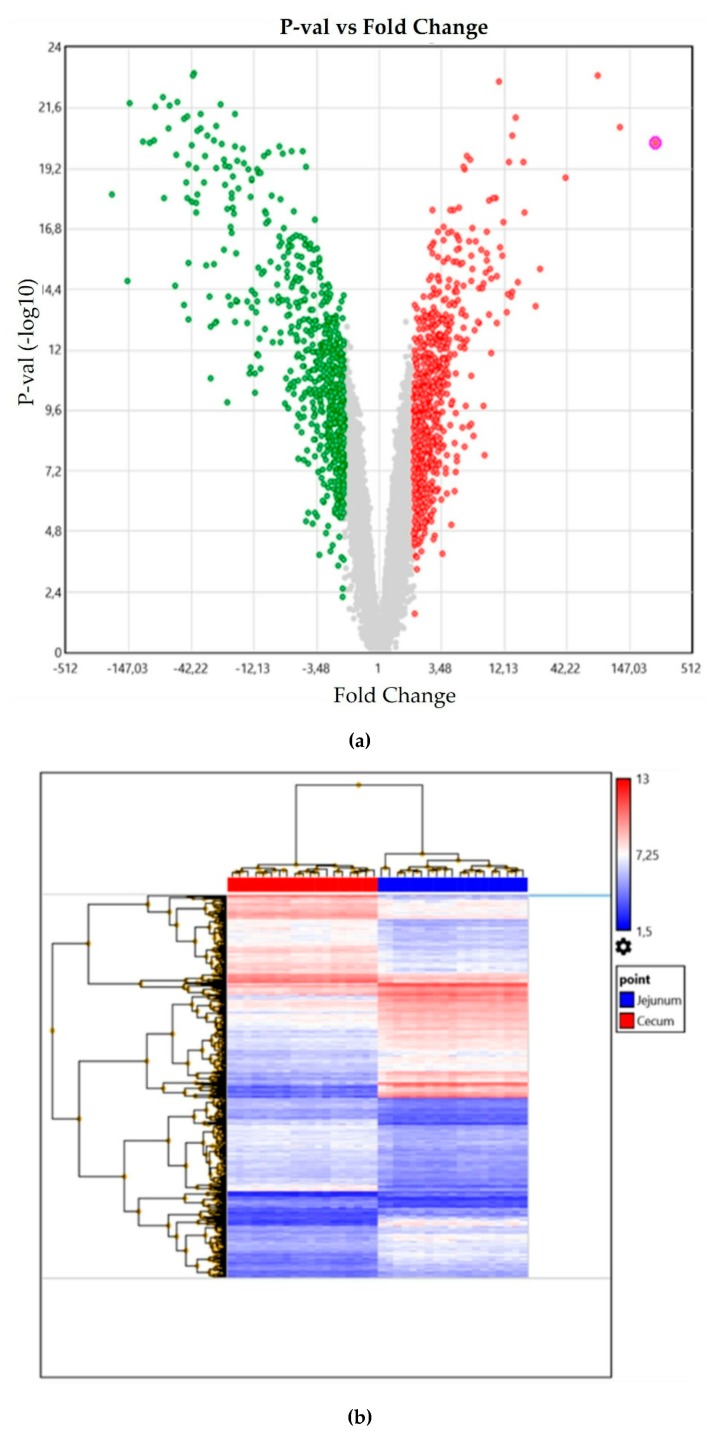
(**a**) Volcano diagram (jejunum in green color, cecum in red color), and (**b**) hierarchical clustering showing the distribution of differentially expressed transcripts (DETs) in the jejunum and cecum of broiler chickens (jejunum in blue color, cecum in red color). Transcripts were considered as DETs when showing a ≥2-fold change ratio (FCR) and a false discovery rate *p*-value (FDR) < 0.05 between tissues.

Data analysis was carried out on the CEL files using the Transcriptomic Analysis Console (TAC) Affymetrix© software (4.0.1.36). Transcripts were considered as differentially expressed transcripts (DETs) when showing a ≥2-fold change ratio (FCR) and a false discovery rate (FDR) <0.05 between tissues. Volcano plots and hierarchical clustering of DETs were obtained by TAC (Figure 1). Transcripts were annotated primarily based on TAC and then using the gene annotation available for *Gallus gallus* (release 85) in Ensembl (www.ensembl.org) [8], based on sequences of Affymetrix probes. Excluding non-protein-coding RNAs, 12,397 genes were recognized. Those genes that were also in the list of DETs were defined as differentially expressed genes (DEGs). The lists of DEGs for the cecum and for the jejunum were submitted to DAVID (Database for Annotation, Visualization, and Integrated Discovery) [9] for functional annotation clustering, and summarization options used were in general Functional_Categories, Gene_Ontology, Pathways, and Protein_Domains. The Kyoto Encyclopedia of Genes and Genomes (KEGG) pathway mapper was used to visualize the pathways more significantly enriched [10].

Gene set analysis was carried out using the Gene Set Enrichment Analysis (GSEA) software based on the C2.CP:KEGG, C5.BP, and C5.MP gene set collections (MSigDB, Broad institute, Cambridge, MA, USA). Normalized enriched score (NES) was calculated for each gene set. Gene sets were considered significantly enriched with false discovery rate (q-value) ≤0.05 and *p*-values of NES <0.05. Transcript data were submitted to the National Center for Biotechnology Information’s Gene Expression Omnibus (NCBI GEO, Bethesda, MD, USA) with the GEO accession number GSE124066. Furthermore, to visualize differences between the jejunum and cecum, the Enrichment Map (http://baderlab.org/Software/EnrichmentMap20) plugin for Cytoscape 3.2.1 (http://www.cytoscape.org) was used to evidence the links between gene sets, considering a node cut-off FDR q-value of 0.10. The nodes were joined if the overlap coefficient was ≥0.4.

## 3. Results

In total, 671 and 681 DETs were found in the jejunum and cecum; of them, 524 and 608 were defined as DEGs.

The lists of the first 20 DETs in the jejunum and cecum, ranked by FCR, are presented in Table 1 and Table 2, respectively, while the full lists for DETs are reported in Tables S2 and S3 (Supplementary Materials). Apolipoprotein B (*APOB*), retinol binding protein 2 (*RBP2*) and glutamyl aminopeptidase (*ENPEP*) in the jejunum, and cystathionine beta-synthase (*CBS*) and C factor like in the cecum were the genes with the highest FCR.

DEGs for the jejunum and cecum were then processed in DAVID and the lists of DAVID functional annotations significantly enriched for DEGs in the jejunum and cecum are presented in Table 3 and Table 4, respectively.

**Figure 2:**
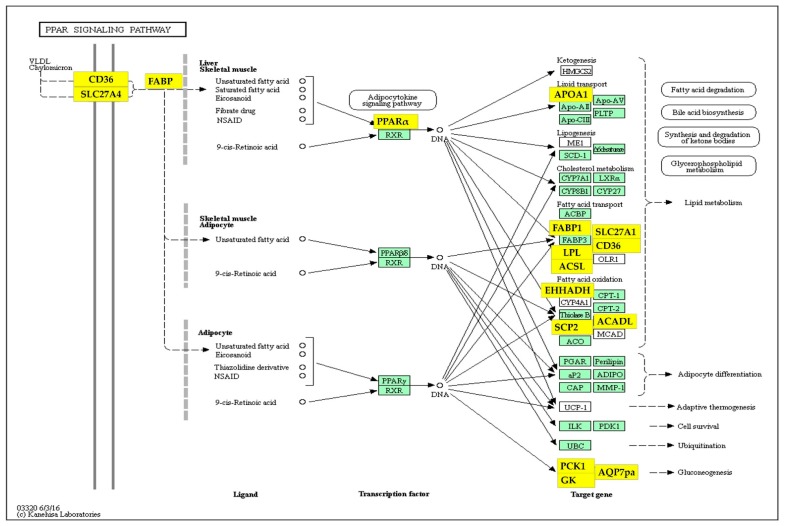
PPAR (peroxisome proliferator-activated receptor) signaling pathway enriched of differentially expressed genes (DEGs) in the jejunum of broiler chickens. The PPAR signaling pathway as the DAVID (Database for Annotation, Visualization and Integrated Discovery) functional annotation significantly enriched DEG is visualized by the Kyoto Encyclopedia of Genes and Genomes (KEGG) [10] pathway mapper. Genes over-expressed in the jejunum are shown in a yellow color. *CD36: CD36* molecule; *ACADL*: acyl-CoA dehydrogenase long chain; *ACSL*: acyl-CoA synthetase long-chain family member (*ACSL3, ACSL4, ACSL5*); *APOA1*: apolipoprotein A-1; *AQP7*: aquaporin 7; *EHHADH:* bi-enzyme enoyl-CoA hydratase/3-hydroxy acyl CoA dehydrogenase; *FABP*: fatty-acid binding protein (*FABP1, FABP2, FABP5, FABP6*); *GK:* glycerol kinase; *LPL*: lipoprotein lipase; *PPARα*: peroxisome proliferator-activated receptor alpha; *PCK1*: phosphoenolpyruvate carboxykinase 1; *SCL27A1/4:* solute carrier family 27 (fatty-acid transporter), member 1, member 4; *SCP2*: solute carrier protein 2.

In the jejunum, most of the DEG-enriched annotations were related to the integral components of the cell membrane (26% of total DEGs) and to the PPAR (peroxisome proliferator-activated receptor) signaling pathway, and peroxisome and lipid metabolism (Table 3). The DEG of the PPAR signaling pathway enriched in the jejunum and the relative link with lipid metabolism are visualized by the KEGG scheme in Figure 2. It is worth signaling that, in addition to those genes expected to control the lipid metabolism, most of those involved in gluconeogenesis were found in the jejunum (*PCK1*, phosphoenolpyruvate carboxykinase 1, soluble, 19.4 FCR; *AQP7*, aquaporin 7, 17.5 FCR; *GK*, glycerol kinase, 2.7 FCR).

**Figure 3:**
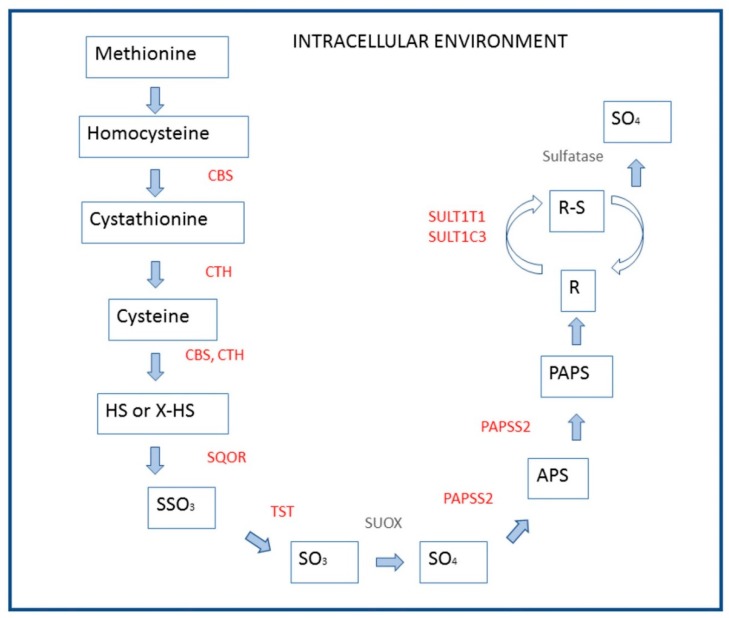
Gene enrichment in the cysteine and methionine catabolism, sulfate metabolism, and disulfide oxidoreductase activity in the cecum of broiler chickens. Genes over-expressed in the cecum with respect to the jejunum are shown in a red color (FDR >2, except *SQOR* (sulfide quinone reductase) at least = 1.8). The alternative sulfate-generating pathway controlled by cysteine dioxygenase type 1 (*CDO1*) is not represented, because the gene coding this enzyme was mildly expressed, with values not differing to jejunum mucosa. *CBS* = cystathionine β-synthase; *CTH* = cystathionine γ-lyase; *SQOR* = sulfide quinone reductase; *TST* = thiosulfate sulfurtransferase; *SUOX* = sulfite oxidase (not spotted on chicken microarrays); *APS* = 5’ adenosine-phosphosulfate; *PAPS* = 3’-phosphoadenosine 5’-phosphosulfate; *PAPSS2* = PAPS synthetase; *SULT1E1* and *SULT1C3* sulfotransferase, family 1C member 3 and family 1E member 1; *GOT2* = glutamic–oxaloacetic transaminase 2; *3-MPY* = 3-mercaptopyruvate; *MPST* = 3-mercaptopyruvate sulfurtransferase; *PY* = pyruvate; *TXN* = thioredoxin. Adapted from Reference [11].

In the cecum, 69 DEGs (11.3% of total DEGs) were related to disulfide bond category, nine to cysteine and methionine metabolism, and 42 DEGs were included in the glycoprotein category. Other enriched categories were related to the cell cycle and extracellular matrix (ECM) (Table 4). The link between cysteine and methionine metabolism, sulfate metabolism, and disulfide oxidoreductase activity, involving several DEGs, is represented in Figure 3. The process starts with the catabolism of methionine and cysteine, more stimulated in the cecum by CBS and cystathionine γ-lyase (*CTH*); then, the DEG signatures involve the “minor” sulfate pathway comprising *CBS, CTH*, sulfide quinone reductase-like (*SQOR*), thiosulfate sulfurtransferase (*TST*), and sulfite oxidase (*SUOX*). The direction toward transport of sulfate is marked by the high FCR of expression of 3’-phosphoadenosine 5’-phosphosulfate synthetase (*PAPSS2*) in the cecum, joined with the high FCR of sulfotransferases *SULT1E1* and *SULT1C3*. Hydrogen sulfide is generated also from cysteine via 3-mercaptopyruvate with joined oxidation of reduced thioredoxin (*TXN*) by 3-mercaptopyruvate sulfurtransferase (*MPST*).

**Figure 4:**
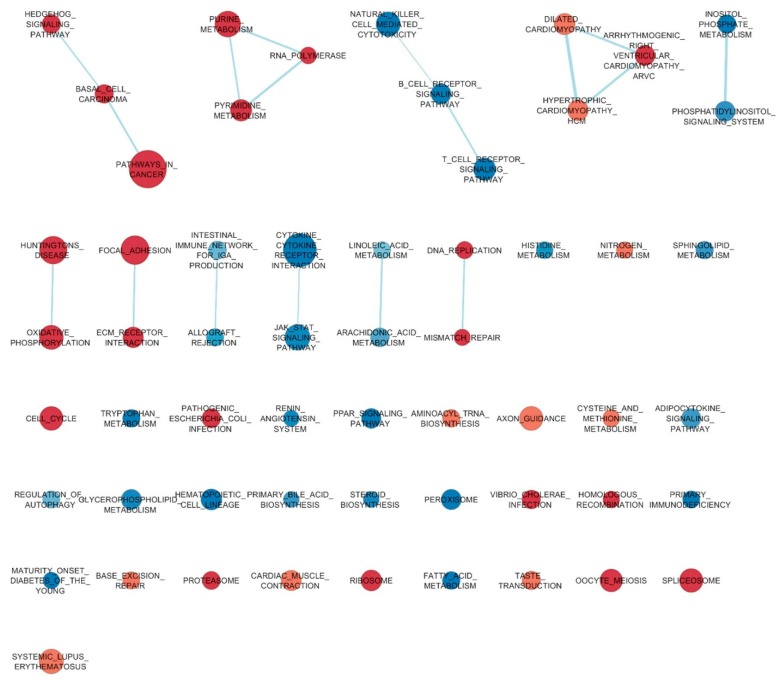
Enriched gene sets in the jejunum and cecum of broiler chickens at 42 days of age. Nodes represent gene sets enriched in the jejunum (red color) and cecum (blue color). Node size represents the number of genes in each gene set. Node cut-off with an FDR q-value of 0.10. The nodes were joined if the overlap coefficient was ≥0.4.

Data of protein-coding gene expression were then analyzed by GSEA that tests the enrichments in predefined sets. Using the KEGG-based list, consisting of 186 gene sets, 14 and 24 gene sets were enriched for the jejunum and cecum, respectively. The full results of this analysis were then processed to create an enrichment map where enrichment sets are eventually linked when sharing relevant numbers of genes. Figure 4 represents the enriched gene sets for the jejunum and cecum according to the KEGG list, while the lists of all the gene sets enriched in the jejunum and cecum are reported in Tables S4 and S5 (Supplementary Materials), respectively. Concerning the jejunum, the top list of gene sets evidenced several gene sets related to the regulation of immunity, particularly concerning IgA production and the tuning of the immune response, as well as peroxisome and PPAR signaling pathway gene sets. Furthermore, the jejunum had an enrichment of the renin–angiotensin system gene set, and of gene sets related to tryptophan and histidine metabolism compared to the cecum. Considering the enrichment analysis performed using the two larger gene aggregates based on Gene Ontology Biological Processes and Molecular Functions, several genes sets related to digestion, absorption, and to bile acid metabolic process (for biological processes), and exo-enzyme and transporter activity (for molecular functions) were among those more enriched in the jejunum. In the cecum, the most enriched gene sets were those related to the cell cycle but also related to the control of the turnover of mature cells, firstly the Hedgehog signaling pathway. There was also the linked ECM receptor interaction and focal adhesion gene sets. Other enriched gene sets were *Vibrio cholerae* infection, pathogenic *Escherichia coli* infection, and taste transduction. In the cecum, the examination of enriched aggregates based on Gene Ontology evidenced a long list of biological processes in general ascribable to the cell cycle and its regulation, as well as to extracellular matrix (ECM) components (laminin binding and extracellular matrix structural constituent) and to disulfide oxidoreductase activities (Table S6, Supplementary Materials).

Non-coding transcripts differentially expressed between the two mucosae were also found: four microRNA (miRNA) and two small RNA (snoRNA) in the jejunum, and four miRNA and one snoRNA were statistically significant, with an FCR ≥2.0 (Table S7, Supplementary Materials).

## 4. Discussion

The small intestine is deputed to digest and absorb nutrients; it is not surprising that it presented enriched gene sets related to secretory enzymes and transporters in comparison with the cecum. Nevertheless, the detailed check of genes with the highest FCR evidenced also some genes already known to be typically present in the end tract of the small intestine, but not often considered for chicken: *RBP2* (151.6 FCR), lactase (*LCT*, 48.6 FCR), cubilin (*CUBN*, intrinsic factor-cobalamin receptor, 44.6 FCR), band eta-carotene 15,15-monooxygenase 1 (*BCMO1*, 11.3 FCR).

Furthermore, the study of pathways evidenced other aspects connected with the absorption of nutrients. The presence of several DEGs related to the renin–angiotensin system (*ENPEP*, 143.7 FCR; angiotensin I converting enzyme 1 and 2, *ACE1* and *ACE2*, with 35.5 and 90.2 FCR, respectively) evidences that the chicken also presents the enterocyte renin–angiotensin system, which in rat was found in brush border, epithelial cells, lamina propria, muscularis mucosa, submucosal blood vessels and muscularis propria [12]. The enterocyte renin–angiotensin system was found to control SGLT1-dependent glucose uptake across the intestinal brush border membrane, sodium and water absorption, and digestion and absorption of peptides [13]. Furthermore, the enterocyte renin–angiotensin system is implicated in the impairment of digestive efficiency in knock-out mice fed an excess of dietary sodium [14].

The enrichment of peroxisome and PPAR signaling-related gene sets, also found as functional annotations based on DEGs by DAVID, was evidently related to the absorption and processing of fats by jejunal enterocytes; however, the inspection of these set evidences also the important presence of a local gluconeogenesis, where glucose is produced and used by the small intestine itself or released into the portal blood [15]. As already previously reported in rat and human [16,17,18], key enzymes and their messenger RNA (mRNA) for gluconeogenesis were found in the small intestine, such as phosphoenolpyruvate carboxykinase (*PEPCK*), one of the two major regulatory genes of gluconeogenesis [15]. In line with previous studies, we found the gene *PCK1* between genes involved in the PPAR signaling pathway, enriched in the jejunum compared to the cecum. The relevance of the small intestine as an endogenous source of glucose and the modulation of this production by the diet in some animals [15,19] and by insulin action [20] were reported, but not for poultry. This activity raises particularly when subjects are underfed or given high-protein diets, since intestinal gluconeogenesis is associated with amino-acid availability [3], whether or not this availability comes from diet or from a long fasting period with the trigger of protein catabolism [21]. Like dietary protein, diets rich in dietary fiber also induce gut gluconeogenesis gene expression and, as well as for amino acids, propionate deriving from the fiber can be used as a precursor [22]. The presence of a portal sensing of intestinal gluconeogenesis in other species [15,23] suggests that intestinal gluconeogenesis in chicken may modulate the interaction between feed characteristics and individual control of feed intake. Finally, the indication that succinate produced by intestinal microbiota activates intestinal gluconeogenesis and, in turn, improves gut homeostasis [24] can be considered as a potential connection of this function with the presence of typical microbial metabolites in chicken, such as lactic acid produced by locally dominant lactic acid bacteria [25].

Sulfur metabolism emerged also as a new key aspect in the comparison between the transcriptomes of the chicken jejunal and cecal tissues. In addition to sulfur amino acids, inorganic sulfate is an essential source for several physiological processes [26]. The high jejunal FCR (32.9) of solute carrier family 13 member 1 (*SLC13A1*, also known as *NaSi*), recognized as the main apical sodium (Na^+^)-dependent transporter into the enterocyte [26], supports that the small intestine is the main site of sulfate absorption in chicken. Interestingly, a several-fold reduced expression of this gene was seen in porcine jejunal loops infused with the enterotoxigenic *Escherichia coli* K88 [27], compared with control loops, indicating that sulfate availability can be impaired by locally induced infection. Moreover, in mice, cecum was recognized as a site of active secretion of sulfates, exchanged for chlorides, by solute carrier family 26 member 3 (*SLC26A3,* also known as *DRA*) [28]; the same transporter *SLC26A3* was upregulated in the cecum (8.1 FCR) and this may indicate that, in chicken, the cecum is also a source of release of sulfates into the lumen. This could also have relevance because sulfates can be used by local microbiota, while it is known that some toxins derived by pathogen bacteria downregulate *SLC26A3* through the increase of intracellular cyclic *AMP* or *GMP* [29] and, conversely, beneficial bacteria upregulated *SLC26A3* gene expression in a CaCo2 cell culture [30] and in pig jejunal loops in vivo [27]. Interestingly, several genes related to sulfur metabolism were upregulated in ceca obtained from chickens conventionally reared or associated with some groups of bacteria, compared to those obtained from germ-free chickens (primarily *CBS, SULT1C3, SULT1E1, TXN, PARSS2,* and *TST*). These genes were related to sulfotransferases and enzymes recognized as sulfate donors; in the cecum, sulfonate groups are widely used both for sulfate conjugation and mucin sulfation for mucus layer building [31]. Since the differences in bacterial community in chickens showed changes in gene expression in sulfur-related genes [31], it may be possible that several pathways and functional associations related to sulfur, which we found in the cecum compared to the jejunum (such as cysteine and methionine metabolism, disulfide bond and disulfide oxidoreductase, activity), are induced or enriched depending on variations in microbiota. The accentuation of these sulfur-related genes role in the cecum may result also from some considerations about the cecal mucin structure. A reduction in sulfomucins in goblet cells in the jejunum, ileum, and colon was associated with the reduced availability of circulating serum sulfates in *SLC13A3* knock-out mice [32]. Mucin sulfation is important to provide the structure, the complexity, and the protection against microbial penetration of mucins [33], which are denser and sulfated in chicken cecum than in jejunum and in chicken than in human [34]. In fact, the sulfated structures detected in the cecum are about 57% of all *O*-glycans compared to the 33% detected in the small intestine [34]. The main enzyme responsible for sulfation of mucin glycans, galactose-3-*O*-sulfotransferase 2 (*GAL3ST2*), was more expressed in the cecum (3.5 FCR) than in the jejunum. However, it worth noting that, for this gene, an extreme variation of individual gene expression was seen here both for the cecum (from 3.5 to 9.7 log_2_ microarray values) and jejunum, as well as in a previous set where two different hybrid genetic lines were compared for gene expression in broiler ilea [35]. Our data cannot allow deciding if this observation is related to a variation of genetic polymorphism in *GAL3ST2* or due to other determinants, including the individual variability of the sulfation of intestinal mucin glycans. Finally, bacteria can release hydrogen sulfide with their metabolic action, thus affecting the intestinal sulfation. Indeed, the balance between dietary organic and inorganic sulfur, endogenous release, and net use by bacteria remains to be assessed. However, more knowledge on these fluxes could also be relevant for methionine and cysteine use by the chicken and to properly appropriate the feeding requirements to the ideal cecum microbiota.

Sulfur is also important for the organization of the ECM; laminin (*LAMB1*) and ECM structural glycoprotein, whose genes were upregulated in the cecum compared to the jejunum (3.4 FCR), contain nearly 200 disulfide bonds [36]. The same was also for a keratan sulfate proteoglycan, lumican (*LUM)*. These genes were included in the enriched gene sets related to ECM structure and formation, and the ECM is important as a structural support, and a biochemical or biomechanical frame for cecum cells. The general observation that pathways related to ECM were enriched for the cecum may indicate that this structure could be considered as a marker of the condition of the local gut barrier in studies aimed at improving this parameter in the chicken cecum. This can imply a revision of sulfur or sulfur amino-acid dietary requirements.

The local metabolism of other amino acids was seen to be affected by the type of intestinal mucosa. In the jejunum, the pathways related to histidine and tryptophan emerged principally. However, the inspection of the list of high-ranking genes for these sets (dopa decarboxylase, aromatic l-amino-acid decarboxylase, monoamine oxidase A, monoamine oxidase B, aldehyde dehydrogenase 3 family member A2) refers to functions (oxidation, decarboxylation) that are not specific to the metabolism of these single amino acids, but rather associated to the use of other essential amino acids. The jejunum is an important site of absorption of amino acids. Thus, it can be considered that a normal portion of amino acids are used locally as a first-pass metabolism, as also seen, for example, in young pig [37]. Conversely, the cecal metabolism depends on nutrients other than essential amino acids, such as those derived from blood; thus, it may activate these pathways to a lesser degree.

The enrichment of several pathways related to immunity in the jejunum is worthy of specific attention. In fact, both sets related to activation (T cells, natural killer cells) and to immune depression were enriched compared to the cecum. The first DEG for the set primary immunodeficiency was adenosine deaminase (*ADA*, FCR = 5.67). Adenosine is a purine nucleoside that is typically released extracellularly, particularly in the case of tissue injuries, and it can be detected by specific cell surface detectors, while it modulates almost all functions of innate and acquired immunity [38]. *ADA* is important because it protects the immune system via adenosine-induced excessive depression, catalyzing the conversion of adenosine to inosine. In fact, combined immunodeficiency diseases can be seen in humans related to genetically derived impaired *ADA* function [39]. Other DEGs were those related to the presence of T cells (CD3D and CD3E) and particularly to cytotoxic types (CD8A). Taking these data together, it indicates that the jejunum requires a more general tuning of the immune system because it is more exposed to offences of bacteria, which may also be due to a lesser defence of the passive barrier compared to the cecum. In support of this, it might be worthwhile considering the enrichment of PPAR signaling in the jejunum from an immunological point of view. In fact, in this study, we identified fatty-acid binding proteins (FABP) between DEGs in the PPAR signaling pathway. FABPs are a class of molecules that not only mediate lipid response and metabolism, but are also closely linked to inflammatory processes. FABPs are involved in the modulation of lipid-sensitive pathways in cells like macrophages, and the importance of FABP presence was suggested for gut barrier health [40]. A decrease in *FABP2* mRNA expression was observed in jejunal mucosa with gut barrier failure in broiler chickens [40]. In our study, *FABP2* was identified as a differentially expressed gene in the jejunum and, thus, in addition to its function in lipid metabolism, it might also be involved in sustaining gut barrier maintenance and defence (more necessary in jejunum that in cecum).

A final consideration should be addressed to the relevant presence of enriched gene sets related to cell cycling and mitosis in the cecum, compared to the jejunum. Both tissues in general have important turnover. However, it is possible that, in chicken, the cecum undergoes more important pressure, including controlled apoptosis to maintain an optimal barrier. This is indicated particularly by the enrichment of the Hedgehog signaling pathway, which is important for the control of large intestinal homeostasis. Interestingly, two of the non-coding transcripts that were also differentially expressed in the cecum (gga-mir-196-4 and gga-mir-1732) are the same cluster on chromosome 2 as the two *Hox* genes, whose differential expression explains principally the effect on the Hedgehog signaling pathway (*HOXA9* and *HOXA10*). A similar cluster is seen also in humans [41], and the integration of miRNA into this system reflects the relevance of certain miRNA to control the expression of gene clusters related to the maturation and maintenance of tissue differentiation in the cecum. Furthermore, it should be considered that the sampled chickens were in a growing phase and, thus, the cecum was still maturing, maybe with a much higher intensity than the jejunum.

## 5. Conclusions

By performing a double exploratory functional analysis on the chicken gut transcriptomic profile, this study confirmed some known and expected intestinal functions, such as those related to nutrient digestion and metabolism and to cell turnover, and it revealed and highlighted new interesting correspondences with mammals not reported before in poultry, such as gluconeogenesis and the renin–angiotensin system in the jejunum. Furthermore, some key aspects emerged from the analysis of DEGs and pathways, indicating a different biological characterization between the different gut sites, which diverge in terms of gene expression toward their main biological processes. In fact, in the jejunum, new key aspects related to sulfur transport activity specific for this site, along with immune pathway tuning, emerged; in the cecum, more intense activity for cell turnover and sulfur utilization for structural components suggest a specific activity at epithelial level, maybe involved in gut barrier maintenance.

Overall, this study may provide potential starting points and hints for future investigations on chicken gut conditions at the molecular level in a broad-based approach on molecular patterns, also considering both the differences between gut tracts and the novel found correspondences with mammals. On the other hand, it would be useful to combine such an exploratory analysis to the analysis of microbiota, including its spatial variations along the gut sites, given its role in the gut balance. Thus, this absence can be a limitation of the study, together with the fact that it was done at a specific age.

## Figures and Tables

**Table 1:** List of the first 20 differentially expressed transcripts (DETs) in the jejunal mucosa of broiler chickens ranked by fold change ratio (FCR), compared to the cecal mucosa.

Fold Change Ratio ^1^	*p*-Value	FDR *p*-Value ^2^	Gene Symbol ^3^	Description
207.3	6.4 × 10^−19^	1.7 × 10^−16^	*APOB*	apolipoprotein B
151.2	1.7 × 10^−15^	1.7 × 10^−13^	*RBP2*	retinol binding protein 2, cellular
143.7	1.5 × 10^−22^	3.8 × 10^−19^	*ENPEP*	glutamyl aminopeptidase (aminopeptidase A)
111.4	5.5 × 10^−21^	4.0 × 10^−18^	*MEP1A*	meprin A, alpha (PABA peptide hydrolase)
98.2	6.0 × 10^−21^	4.2 × 10^−18^	*SI*	sucrase-isomaltase (alpha-glucosidase)
90.2	4.9 × 10^−21^	3.7 × 10^−18^	*ACE2*	angiotensin I converting enzyme 2
88.0	2.2 × 10^−22^	4.1 × 10^−19^	*SLC6A19*	solute carrier family 6 (neutral amino acid transporter), member 19
74.2	9.2 × 10^−23^	3.4 × 10^−19^	*MGAM*	maltase-glucoamylase (alpha-glucosidase)
73.2	9.5 × 10^−19^	2.3 × 10^−16^	*SLC7A9*	solute carrier family 7 (amino acid transporter light chain, bo, +system), member 9
66.3	1.5 × 10^−21^	1.6 × 10^−18^	*SLC15A1*	oligopeptide transporter, member 1
64.8	1.9 × 10^−22^	3.8 × 10^−19^	*SLC9A3*	(NHE3, cation proton antiporter 3), member 3
58.4	2.9 × 10^−15^	2.7 × 10^−13^	*ENPP7*	ectonucleotide pyrophosphatase/phosphodiesterase 7
57.9	1.7 × 10^−20^	8.3 × 10^−18^	*CLDN10*	claudin 10
55.6	1.4 × 10^−22^	3.8 × 10^−19^	*MGAT4D*	mannosyl (alpha-1,3-)-glycoprotein beta-1,4-*N*-acetylglucosaminyltransferase, isozyme B-like
49.6	1.6 × 10^−14^	1.2 × 10^−12^	*LCT*	Lactase
49.2	6.6 × 10^−22^	8.0 × 10^−19^	*CNOT2*	CCR4-NOT transcription complex, subunit 2
46.6	2.3 × 10^−19^	6.6 × 10^−17^	*TM4SF4*	transmembrane 4 L six family member 4
46.3	9.6 × 10^−19^	2.3 × 10^−16^	*MME*	membrane metallo-endopeptidase
45.8	5.1 × 10^−22^	7.1 × 10^−19^	*MEP1B*	meprin A, beta
45.0	3.7 × 10^−16^	4.3 × 10^−14^	*FABP2*	Fatty-acid binding protein 2, intestinal

^1,2^ Affymetrix transcripts were considered as differentially expressed transcripts (DETs) when showing a ≥2-fold change ratio (FCR) and a false discovery rate (FDR) < 0.05 between tissues. ^3^ Transcripts were annotated based on *Gallus gallus* Ensembl (release 85, www.ensembl.org). The full list of differentially expressed genes is reported in Table S2 (Supplementary Materials).

**Table 2:** List of the first 20 differentially expressed transcripts in cecum of broiler chickens ranked by fold change ratio (FCR), compared to the jejunal mucosa.

Fold Change Ratio ^1^	*p*-Value	FDR *p*-Value ^2^	Gene Symbol ^3^	Description
244.4	6.17 × 10^−21^	4.18 × 10^−18^	*CBS*	cystathionine-beta-synthase
121.2	1.44 × 10^−21^	1.55 × 10^−18^	*ENSGALG00000021450*	C factor like
78.4	1.19 × 10^−23^	7.73 × 10^−20^	*MAL*	mal, T-cell differentiation protein
40.8	1.42 × 10^−19^	4.34 × 10^−17^	*AQP8*	aquaporin 8
24.5	6.06 × 10^−16^	6.60 × 10^−14^	*NOXO1*	NADPH oxidase organizer 1
22.5	1.76 × 10^−14^	1.24 × 10^−12^	*CA4*	carbonic anhydrase IV
18.0	3.58 × 10^−18^	7.28 × 10^−16^	*HOXA10*	homeobox A10; homeobox protein Hox-A10-like
17.9	3.44 × 10^−20^	1.40 × 10^−17^	*SLC38A4*	solute carrier family 38, member 4 (SNAT4)
15.8	2.11 × 10^−15^	2.01 × 10^−13^	*SLC26A4*	solute carrier family 26 (anion exchanger), member 4
15.1	5.97 × 10^−22^	7.80 × 10^−19^	*PON2*	paraoxonase 2
14.2	4.79 × 10^−15^	4.14 × 10^−13^	*TFCP2L1*	transcription factor CP2-like 1
14.2	2.93 × 10^−21^	2.55 × 10^−18^	*SELENBP1*	selenium binding protein 1; selenium-binding protein 1-A-like
14.0	7.89 × 10^−15^	6.28 × 10^−13^	*ATP6V0D2*	ATPase, H+ transporting, lysosomal 38 kDa, V0 subunit d2
13.2	3.34 × 10^−20^	1.39 × 10^−17^	*PADI3*	peptidyl arginine deiminase, type III
12.9	5.97 × 10^−15^	4.99 × 10^−13^	*PLET1*	Placenta expressed transcript 1
12.9	3.03 × 10^−14^	2.05 × 10^−12^	*GJB2*	gap junction protein, beta 2, 26 kDa
12.0	8.16 × 10^−18^	1.57 × 10^−15^	*LY6E*	lymphocyte antigen 6 complex, locus E-like
11.7	1.74 × 10^−16^	2.28 × 10^−14^	*gga-mir-196-4*	microRNA 196-4
11.2	8.74 × 10^−17^	1.25 × 10^−14^	*GSTA4*	glutathione *S*-transferase alpha 4
11.0	2.26 × 10^−23^	1.03 × 10^−19^	*B4GALNT3*	beta-1,4-*N*-acetyl-galactosaminyltransferase 3

^1,2^ Affymetrix transcripts were considered as differentially expressed transcripts (DETs) when showing a ≥2-fold change ratio (FCR) and a false discovery rate (FDR) < 0.05 between tissues. ^3^ Transcripts were annotated based on *Gallus gallus* Ensembl (release 85, www.ensembl.org). The full list of differentially expressed genes is reported in Table S3 (Supplementary Materials).

**Table 3:** List of DAVID (Database for Annotation, Visualization and Integrated Discovery) functional annotations significantly enriched of differentially expressed genes (DEGs) in the jejunal mucosa of broiler chickens.

Categories and Functional Annotations ^1^	% ^2^	*p*-Value ^1^	Benjamini Value ^1^
KEGG_PATHWAY			
PPAR signaling pathway	3.4	9 × 10^−11^	9 × 10^−9^
Metabolic pathways	14.9	3 × 10^−8^	1 × 10^−6^
Peroxisome	2.3	3 × 10^−4^	1 × 10^−2^
Glycerophospholipid metabolism	2.3	1 × 10^−3^	3 × 10^−2^
Histidine metabolism	1.1	1 × 10^−3^	3 × 10^−2^
Fatty acid degradation	1.3	2 × 10^−3^	4 × 10^−2^
UP_KEYWORDS			
Transmembrane helix	33.0	1 × 10^−11^	3 × 10^−9^
Transmembrane	33.0	2 × 10^−11^	2 × 10^−9^
Membrane	34.2	9 × 10^−10^	6 × 10^−8^
Transport	7.4	5 × 10^−4^	2 × 10^−2^
GOTERM_BP_DIRECT			
Cholesterol efflux	1.3	2 × 10^−5^	3 × 10^−2^
GOTERM_CC_DIRECT			
Apical plasma membrane	3.8	2 × 10^−8^	5 × 10^−6^
Integral component of membrane	26.3	3 × 10^−7^	3 × 10^−5^
Brush border membrane	1.5	1 × 10^−5^	7 × 10^−4^
Peroxisome	1.9	9 × 10^−5^	4 × 10^−3^

^1^ Protocol of DAVID functional analysis, as described by Reference [9]. ^2^ Percentages of the total differentially expressed genes (DEGs) obtained from DAVID analysis.

**Table 4:** List of DAVID (Database for Annotation, Visualization and Integrated Discovery) functional annotation significantly enriched of differentially expressed genes (DEGs) in the cecal mucosa of broiler chickens.

Categories and Functional Annotations ^1^	% ^2^	*p*-Value ^1^	Benjamini Value ^1^
KEGG_PATHWAY			
Cysteine and methionine metabolism	1.5	0.0001	0.012
Cell cycle	2.6	0.0001	0.009
Focal adhesion	3.3	0.0008	0.032
UP_KEYWORDS			
Disulfide bond	11.3	0.0000	0.002
Mitosis ^3^	2.0	0.0000	0.001
Developmental protein	4.8	0.0000	0.002
Glycoprotein	6.9	0.0000	0.002
ATP-binding	7.9	0.0002	0.008
Secreted	5.8	0.0002	0.007
Alternative splicing	3.0	0.0003	0.008
Cytoskeleton	2.8	0.0016	0.030
Phosphoprotein	4.3	0.0033	0.053
GOTERM_BP_DIRECT			
Chromosome segregation	1.6	0.0000	0.044
GOTERM_CC_DIRECT			
Proteinaceous extracellular matrix ^4^	3.9	0.0000	0.000
Midbody ^5^	2.0	0.0001	0.013
Kinesin complex ^6^	1.5	0.0002	0.012
GOTERM_MF_DIRECT			
Heparin binding	2.5	0.0000	0.001
Chemoattractant activity	1.2	0.0001	0.022

^1^ Protocol of DAVID functional analysis, as described by Reference [9]. ^2^ Percentages of the total differentially expressed genes (DEGs) obtained from DAVID analysis. ^3,4,5,6^ Other categories statistically significant: ^3^ cell division, centromere, cell cycle, nucleotide binding, DNA binding, chromosome; ^4^ extracellular exosome, extracellular space, focal adhesion; ^5^ spindle microtubule; ^6^ kinetocore, condensed chromosome kinetochore.

## Supplementary Materials

The following are available online at https://www.mdpi.com/2076-2615/9/5/221/s1: Table S1: Composition of the commercial diets; Table S2: List of the assigned differentially expressed transcripts in jejunal mucosa of broiler chickens ranked for the fold change ratio (FCR), compared to cecal mucosa; Table S3: List of the assigned differentially expressed transcripts in cecal mucosa of broiler chickens ranked for the fold change ratio (FCR), compared to jejunal mucosa; Table S4: Gene sets enriched in jejunal mucosa of broiler chickens, compared to cecal mucosa, ranked for the fold change ratio (FDR), q-value ≤0.05; Table S5: Gene sets enriched in cecal mucosa of broiler chickens, compared to jejunal mucosa, ranked for the fold change ratio (FDR), q-value ≤0.05; Table S6: The first 20 gene sets of Gene Ontology Biological Processes-derived list enriched in cecal mucosa of broiler chickens, compared to jejunal mucosa, ranked for the fold change ration (FDR), q-value ≤0.05; Table S7: Non-coding differentially expressed transcripts in jejunal or cecal mucosa of broiler chickens, with a log_2_-fold change ratio (FCR) ≥2.
