# Supplementary material for: Exploring Differential Transcriptome between Jejunal and Cecal Tissue of Broiler Chickens
Source: Animals (Basel). 2019 May 7;9(5):221. doi: 10.3390/ani9050221 (PMC6562892; doi:10.3390/ani9050221)
Supplement: Supplementary file 1 [file animals-09-00221-s001.zip › supplementary files/Table S 1.docx]

**Supplementary Table 1**. Composition of the commercial diets**.**

| Item | STARTER  (0-10 d) | GROWER 1  (11-25 d) | FINISHER  (26-42 d) |
| --- | --- | --- | --- |
|  |  |  |  |
| Corn | 42.17 | 34.96 | 12.73 |
| White Corn | 0.00 | 0.00 | 15.00 |
| Wheat | 10.00 | 20.00 | 25.01 |
| Sorghum | 0.00 | 0.00 | 5.00 |
| Soybean Meal | 23.11 | 20.63 | 17.60 |
| Expanded Soybean | 10.00 | 10.00 | 13.00 |
| Sunflower | 3.00 | 3.00 | 3.00 |
| Corn Gluten | 4.00 | 3.00 | 0.00 |
| Soybean Oil | 3.08 | 4.43 | 5.48 |
| Dicalcium phosphate | 1.52 | 1.20 | 0.57 |
| Calcium carbonate | 0.91 | 0.65 | 0.52 |
| Sodium bicarbonate | 0.15 | 0.10 | 0.15 |
| Salt | 0.27 | 0.27 | 0.25 |
| Choline chloride | 0.10 | 0.10 | 0.10 |
| Lysine sulphate | 0.59 | 0.55 | 0.46 |
| Dl-Methionine | 0.27 | 0.29 | 0.30 |
| Threonine | 0.15 | 0.14 | 0.14 |
| Enzyme - Roxazyme G2G | 0.08 | 0.08 | 0.08 |
| Phytase 0.1% | 0.10 | 0.10 | 0.10 |
| Vitamin - Mineral Premix^1^ | 0.50 | 0.50 | 0.50 |
|  |  |  |  |
| Dry Matter,% | 88.57 | 88.65 | 88.64 |
| Protein,% | 22.70 | 21.49 | 19.74 |
| Lipid,% | 7.06 | 8.24 | 9.74 |
| Fibre,% | 3.08 | 3.04 | 3.07 |
| Ash,% | 5.85 | 5.17 | 4.49 |
| Lysine,% | 1.38 | 1.29 | 1.21 |
| Methionine,% | 0.67 | 0.62 | 0.59 |
| Methionine + Cysteine,% | 1.03 | 0.97 | 0.91 |
| Calcium,% | 0.91 | 0.80 | 0.59 |
| Phosphate,% | 0.63 | 0.57 | 0.46 |
| Metabolizable Energy (Kcal/Kg) | 3.076 | 3.168 | 3.264 |

^1^Provided the following per kg of diet: vitamin A (retinyl acetate), 13,000 IU; vitamin D3 (cholecalciferol), 4,000 IU; vitamin E (DL-α_tocopheryl acetate), 80 IU; vitamin K (menadione sodium bisulfite), 3 mg; riboflavin, 6.0 mg; pantothenic acid, 6.0 mg; niacin, 20 mg; pyridoxine, 2 mg; folic acid, 0.5 mg; biotin, 0.10 mg; thiamine, 2.5 mg; vitamin B12 20 μg; Mn, 100 mg; Zn, 85 mg; Fe, 30 mg; Cu, 10 mg; I, 1.5 mg; Se, 0.2 mg; ethoxyquin, 100 mg.
