# Supplementary material for: Exploring Differential Transcriptome between Jejunal and Cecal Tissue of Broiler Chickens
Source: Animals (Basel). 2019 May 7;9(5):221. doi: 10.3390/ani9050221 (PMC6562892; doi:10.3390/ani9050221)
Supplement: Supplementary file 1 [file animals-09-00221-s001.zip › supplementary files/Table S 2.docx]

**Supplementary Table 2**. List of the assigned differentially expressed transcripts in jejunal mucosa of broiler chickens ranked for the fold change ratio (FCR), compared to cecal mucosa.

| Fold Change^1^ | *P*-value | FDR  *P*-value^2^ | Gene  Symbol^3^ | Description |
| --- | --- | --- | --- | --- |
| 207.3 | 6.39E-19 | 1.700E-16 | APOB | Apolipoprotein B x chilomicroni e LDL |
| 151.2 | 1.73E-15 | 1.690E-13 | RBP2 | retinol binding protein 2, cellular |
| 143.7 | 1.52E-22 | 3.810E-19 | ENPEP | glutamyl aminopeptidase (aminopeptidase A) |
| 111.4 | 5.45E-21 | 3.990E-18 | MEP1A | meprin A, alpha (PABA peptide hydrolase) |
| 98.2 | 6.01E-21 | 4.180E-18 | SI | sucrase-isomaltase (alpha-glucosidase) |
| 90.2 | 4.89E-21 | 3.730E-18 | ACE2 | angiotensin I converting enzyme 2 |
| 88.0 | 2.24E-22 | 4.100E-19 | SLC6A19 | solute carrier family 6 (neutral amino acid transporter), member 19 |
| 74.2 | 9.15E-23 | 3.350E-19 | MGAM | maltase-glucoamylase (alpha-glucosidase) |
| 73.2 | 9.46E-19 | 2.300E-16 | SLC7A9 | solute carrier family 7 (amino acid transporter light chain, bo,+ system), member 9 |
| 66.3 | 1.53E-21 | 1.550E-18 | SLC15A1 | (oligopeptide transporter), member 1 |
| 64.8 | 1.87E-22 | 3.810E-19 | SLC9A3 | (NHE3, cation proton antiporter 3), member 3 |
| 58.4 | 2.93E-15 | 2.680E-13 | ENPP7 | ectonucleotide pyrophosphatase/phosphodiesterase 7 |
| 57.9 | 1.69E-20 | 8.340E-18 | CLDN10 | claudin 10 |
| 55.6 | 1.38E-22 | 3.810E-19 | MGAT4D | mannosyl (alpha-1,3-)-glycoprotein beta-1,4-N-acetylglucosaminyltransferase, isozyme B-like |
| 49.6 | 1.61E-14 | 1.150E-12 | LCT | Lactase |
| 49.2 | 6.58E-22 | 8.040E-19 | CNOT2 | CCR4-NOT transcription complex, subunit 2 |
| 46.6 | 2.31E-19 | 6.600E-17 | TM4SF4 | transmembrane 4 L six family member 4 |
| 46.3 | 9.57E-19 | 2.300E-16 | MME | membrane metallo-endopeptidase |
| 45.9 | 5.06E-22 | 7.120E-19 | MEP1B | meprin A, beta |
| 45.0 | 3.66E-16 | 4.330E-14 | FABP2 | fatty acid binding protein 2, intestinal |
| 44.9 | 4.05E-20 | 1.580E-17 | MALRD1 | MAM And LDL Receptor Class A Domain Containing 1 |
| 44.6 | 6.22E-14 | 3.800E-12 | CUBN | cubilin (intrinsic factor-cobalamin receptor) |
| 42.3 | 1.27E-18 | 2.940E-16 | PLA2G2E | phospholipase A2, group IIE |
| 40.7 | 1.27E-23 | 7.730E-20 | ABCG5 | ATP-binding cassette, sub-family G (WHITE), member 5 |
| 40.5 | 1.01E-23 | 7.730E-20 | PDZK1 | PDZ domain containing 1 |
| 38.8 | 3.33E-18 | 6.850E-16 | CYP2K1L | cytochrome P450 2K1-like |
| 38.4 | 1.47E-18 | 3.360E-16 | LEAP2 | liver expressed antimicrobial peptide 2 |
| 37.9 | 1.97E-21 | 1.810E-18 | ABCG8 | ATP-binding cassette, sub-family G member 8 |
| 37.3 | 6.36E-19 | 1.700E-16 | CPO | carboxypeptidase O |
| 35.5 | 1.61E-21 | 1.550E-18 | ACE | angiotensin I converting enzyme |
| 35.0 | 4.12E-22 | 6.290E-19 | CLIC5 | chloride intracellular channel 5 |
| 32.9 | 2.11E-19 | 6.130E-17 | SLC13A1 | solute carrier family 13 (sodium/sulfate symporter), member 1 |
| 32.0 | 1.88E-20 | 8.640E-18 | SLC3A1 | solute carrier family 3 (amino acid transporter heavy chain), member 1 |
| 31.5 | 4.13E-16 | 4.700E-14 | APOA1 | apolipoprotein A-I |
| 30.6 | 3.13E-21 | 2.610E-18 | GCG | Glucagon |
| 29.5 | 7.67E-15 | 6.160E-13 | AGMO | alkylglycerol monooxygenase |
| 29.0 | 1.19E-13 | 6.450E-12 | FABP6 | fatty acid binding protein 6 |
| 27.3 | 4.05E-16 | 4.640E-14 | CYP2C23b | cytochrome P450 2H1 |
| 26.9 | 4.77E-21 | 3.730E-18 | MTTP | microsomal triglyceride transfer protein |
| 26.6 | 8.24E-14 | 4.670E-12 | SLC2A2 | solute carrier family 2 (facilitated glucose transporter), member 2 |
| 26.1 | 7.66E-14 | 4.420E-12 | SLC26A9 | solute carrier family 26 (anion exchanger), member 9 |
| 23.6 | 1.67E-22 | 3.810E-19 | GATA5 | GATA binding protein 5 |
| 23.2 | 6.39E-21 | 4.180E-18 | FRMPD2 | FERM and PDZ domain containing 2 |
| 22.1 | 1.09E-16 | 1.480E-14 | TMEM86A | transmembrane protein 86 A |
| 21.9 | 4.63E-20 | 1.770E-17 | ST3GAL5 | ST3 Beta-Galactoside Alpha-2,3-Sialyltransferase 5 |
| 21.6 | 2.77E-19 | 7.800E-17 | FUT9 | fucosyltransferase 9 (alpha (1,3) fucosyltransferase) |
| 20.9 | 1.23E-10 | 2.720E-09 | TMPRSS15 | transmembrane protease, serine 15 |
| 20.6 | 2.53E-18 | 5.590E-16 | ALDOB | aldolase B, fructose-bisphosphate |
| 20.2 | 7.71E-15 | 6.170E-13 | ACOT12 | acyl-CoA thioesterase 12 |
| 19.8 | 5.21E-20 | 1.950E-17 | CYP2D6 | cytochrome P450, family 2, subfamily D, polypeptide 6 |
| 19.4 | 1.33E-17 | 2.480E-15 | PCK1 | phosphoenolpyruvate carboxykinase 1 (soluble) |
| 19.2 | 1.62E-14 | 1.160E-12 | ASAH2 | N-acylsphingosine amidohydrolase (non-lysosomal ceramidase) 2 |
| 19.1 | 8.97E-19 | 2.230E-16 | SLC2A5 | solute carrier family 2 (facilitated glucose/fructose transporter), member 5 |
| 19.0 | 6.05E-19 | 1.650E-16 | SLC1A1 | solute carrier family 1 (neuronal/epithelial high affinity glutamate transporter, system Xag), member 1 |
| 18.9 | 2.23E-17 | 3.890E-15 | TTLL2 | tubulin tyrosine ligase-like family member 2 |
| 18.7 | 1.34E-19 | 4.140E-17 | PLEKHS1 | pleckstrin homology domain containing, family S member 1 |
| 18.5 | 2.13E-18 | 4.800E-16 | MFI2 | antigen p97 (melanoma associated) identified by monoclonal antibodies 133.2 and 96.5 |
| 18.0 | 3.70E-18 | 7.380E-16 | HKDC1 | hexokinase domain containing 1 |
| 17.7 | 8.24E-21 | 4.870E-18 | SLC5A1 | solute carrier family 5 (sodium/glucose cotransporter), member 1 |
| 17.7 | 4.06E-22 | 6.290E-19 | HNF4B | hepatocyte nuclear factor 4 beta |
| 17.6 | 7.58E-14 | 4.420E-12 | SCT | Secretin |
| 17.5 | 9.93E-15 | 7.740E-13 | AQP7 | aquaporin 7 |
| 17.4 | 1.15E-14 | 8.640E-13 | IYD | iodotyrosine deiodinase |
| 16.9 | 1.12E-14 | 8.430E-13 | GATA4 | GATA binding protein 4 |
| 16.6 | 3.93E-19 | 1.090E-16 | ALPP | alkaline phosphatase, placental |
| 15.1 | 1.03E-20 | 5.880E-18 | ABCC2 | ATP-binding cassette, sub-family C (CFTR/MRP), member 2 |
| 13.9 | 8.20E-14 | 4.660E-12 | APOA4 | apolipoprotein A4 |
| 13.6 | 6.46E-20 | 2.190E-17 | NTS | Neurotensin |
| 13.3 | 8.08E-12 | 2.510E-10 | SLC5A11 | solute carrier family 5 (sodium/inositol cotransporter), member 11, |
| 12.9 | 4.70E-12 | 1.590E-10 | CALB1 | calbindin 1, 28kDa |
| 12.8 | 4.14E-15 | 3.620E-13 | ANO5 | anoctamin 5 |
| 12.6 | 2.00E-14 | 1.390E-12 | CYP4V2 | cytochrome P450, family 4, subfamily V, polypeptide 2 |
| 12.0 | 5.13E-11 | 1.260E-09 | ETNPPL | ethanolamine-phosphate phospho-lyase |
| 11.9 | 6.28E-15 | 5.200E-13 | GIP | gastric inhibitory polypeptide |
| 11.9 | 8.32E-12 | 2.570E-10 | GLUL | glutamate-ammonia ligase |
| 11.6 | 3.06E-14 | 2.060E-12 | TMEM252 | transmembrane protein 252 |
| 11.5 | 1.21E-14 | 8.880E-13 | GIMD1 | GIMAP family P-loop NTPase domain containing 1 |
| 11.5 | 6.73E-20 | 2.200E-17 | SLC22A13L | solute carrier family 22 member 13-like |
| 11.3 | 1.53E-12 | 5.830E-11 | BCMO1 | beta-carotene 15,15-monooxygenase 1 |
| 11.2 | 8.94E-20 | 2.840E-17 | SLC5A9 | solute carrier family 5 (sodium/sugar cotransporter), member 9 |
| 11.2 | 5.58E-16 | 6.240E-14 | CYP3A7 | cytochrome P450 A 37 |
| 11.1 | 1.72E-12 | 6.480E-11 | SLC34A2 | solute carrier family 34 (type II sodium/phosphate contransporter), member 2 |
| 10.8 | 3.66E-13 | 1.710E-11 | SUSD2 | sushi domain containing 2 |
| 10.8 | 5.52E-12 | 1.820E-10 | SLC5A12 | solute carrier family 5 (sodium/monocarboxylate cotransporter), member 12 |
| 10.6 | 9.00E-20 | 2.840E-17 | GDA | guanine deaminase |
| 9.9 | 7.50E-16 | 7.990E-14 | REEP6 | receptor accessory protein 6 |
| 9.9 | 1.87E-20 | 8.640E-18 | GPD1L2 | glycerol-3-phosphate dehydrogenase 1-like 2 |
| 9.8 | 2.98E-18 | 6.190E-16 | AKR1D1 | aldo-keto reductase family 1, member D1 (delta 4-3-ketosteroid-5-beta-reductase) |
| 9.8 | 1.47E-13 | 7.720E-12 | SCARB1 | scavenger receptor class B, member 1 |
| 9.6 | 3.71E-18 | 7.380E-16 | ABCB1LB | ATP-binding cassette, sub-family B (MDR/TAP), member 1-like B |
| 9.5 | 9.13E-18 | 1.740E-15 | RNF128 | ring finger protein 128 |
| 9.2 | 7.41E-18 | 1.440E-15 | SLC28A2 | solute carrier family 28 (sodium-coupled nucleoside transporter), member 2 |
| 9.1 | 1.35E-20 | 7.060E-18 | SH2D4A | SH2 domain containing 4° |
| 8.7 | 2.97E-16 | 3.610E-14 | SLC19A3 | solute carrier family 19, member 3 |
| 8.6 | 1.04E-14 | 8.000E-13 | GPR128 | G protein-coupled receptor 128 |
| 8.5 | 7.19E-19 | 1.880E-16 | DPP4 | dipeptidyl-peptidase 4 |
| 8.3 | 4.78E-13 | 2.140E-11 | ENPP3 | ectonucleotide pyrophosphatase/phosphodiesterase 3 |
| 7.7 | 2.54E-15 | 2.370E-13 | CYBRD1 | cytochrome b reductase 1 |
| 7.5 | 5.59E-16 | 6.240E-14 | HMCN1 | hemicentin 1 |
| 7.5 | 1.29E-14 | 9.430E-13 | ADH6 | alcohol dehydrogenase 6 (class V) |
| 7.4 | 7.28E-15 | 5.900E-13 | GGT1 | gamma-glutamyltransferase 1 |
| 7.3 | 7.67E-17 | 1.120E-14 | CREB3L3 | cAMP responsive element binding protein 3-like 3 |
| 7.3 | 3.11E-17 | 5.230E-15 | SORD | sorbitol dehydrogenase |
| 7.3 | 8.00E-21 | 4.870E-18 | KLB | klotho beta |
| 7.2 | 1.93E-13 | 9.790E-12 | RBP1 | retinol binding protein 1, cellular |
| 7.2 | 1.07E-18 | 2.540E-16 | HSD11B1b | hydroxysteroid (11-beta) dehydrogenase 1b |
| 7.1 | 4.81E-13 | 2.150E-11 | GRAMD3 | GRAM domain containing 3 |
| 7.1 | 5.64E-17 | 8.670E-15 | GPT2 | glutamic pyruvate transaminase (alanine aminotransferase) 2 |
| 6.9 | 1.08E-14 | 8.180E-13 | PLA2G12B | phospholipase A2, group XIIB |
| 6.8 | 1.59E-20 | 8.090E-18 | DAB1 | Dab, reelin signal transducer, homolog 1 (Drosophila) |
| 6.7 | 1.49E-17 | 2.730E-15 | SOX6 | SRY (sex determining region Y)-box 6 |
| 6.7 | 2.12E-16 | 2.670E-14 | GDPD4 | glycerophosphodiester phosphodiesterase domain containing 4 |
| 6.4 | 1.31E-11 | 3.850E-10 | TMEM56 | transmembrane protein 56 |
| 6.3 | 7.47E-16 | 7.990E-14 | CDKL2 | cyclin-dependent kinase-like 2 (CDC2-related kinase) |
| 6.3 | 3.83E-16 | 4.470E-14 | CAT | catalase |
| 6.3 | 4.41E-14 | 2.830E-12 | NMNAT3 | nicotinamide nucleotide adenylyltransferase 3 |
| 6.2 | 1.00E-14 | 7.750E-13 | EPHX1 | epoxide hydrolase 1, microsomal (xenobiotic) |
| 6.2 | 1.29E-15 | 1.310E-13 | CES2 | carboxylesterase 2 (fatty acyl-CoA hydrolase precursor, medium chain) |
| 6.2 | 7.24E-17 | 1.070E-14 | B3GNT5 | UDP-GlcNAc:betaGal beta-1,3-N-acetylglucosaminyltransferase 5 |
| 6.1 | 1.34E-16 | 1.800E-14 | PHOSPHO1 | phosphatase, orphan 1 |
| 6.0 | 1.78E-14 | 1.250E-12 | TMC5 | transmembrane channel-like 5 |
| 5.9 | 1.95E-13 | 9.860E-12 | GLOD5 | glyoxalase domain containing 5 |
| 5.8 | 2.28E-11 | 6.280E-10 | MAOB | monoamine oxidase B |
| 5.8 | 6.62E-12 | 2.120E-10 | XKR9 | XK, Kell blood group complex subunit-related family, member 9 |
| 5.8 | 1.24E-20 | 6.860E-18 | TRIM36 | tripartite motif containing 36 |
| 5.7 | 1.17E-13 | 6.370E-12 | SLC16A9 | solute carrier family 16, member 9 |
| 5.7 | 7.72E-17 | 1.120E-14 | FMO4 | flavin containing monooxygenase 4 |
| 5.7 | 3.33E-17 | 5.550E-15 | ADA | adenosine deaminase |
| 5.6 | 2.31E-15 | 2.180E-13 | SEMA5B | sema domain, seven thrombospondin repeats (type 1 and type 1-like), transmembrane domain (TM) and short cytoplasmic domain, (semaphorin) 5B |
| 5.5 | 2.94E-18 | 6.190E-16 | TBC1D24 | TBC1 Domain Family Member 24: Vesicle-mediated transport and GTPase activator activity |
| 5.3 | 1.70E-15 | 1.660E-13 | CUTA | cutA divalent cation tolerance homolog (E. coli) |
| 5.3 | 1.29E-15 | 1.310E-13 | MAFB | MAF bZIP transcription factor B |
| 5.3 | 1.27E-14 | 9.280E-13 | A1CF | APOBEC1 complementation factor |
| 5.3 | 2.90E-17 | 4.960E-15 | ENPP6 | ectonucleotide pyrophosphatase/phosphodiesterase 6 |
| 5.2 | 2.58E-13 | 1.260E-11 | FAM83B | family with sequence similarity 83, member B |
| 5.2 | 3.63E-13 | 1.710E-11 | CL2 | liver ribonuclease A |
| 5.2 | 1.82E-16 | 2.370E-14 | EPHX1L | epoxide hydrolase 1-like |
| 5.1 | 4.44E-13 | 2.040E-11 | RMDN2 | regulator of microtubule dynamics 2 |
| 5.1 | 1.77E-12 | 6.630E-11 | snoRNA RF00004 |  |
| 5.1 | 1.95E-14 | 1.370E-12 | KCNE2 | potassium voltage-gated channel, Isk-related family, member 2 |
| 5.0 | 1.54E-12 | 5.850E-11 | FBP1 | fructose-1,6-bisphosphatase 1 |
| 5.0 | 3.51E-10 | 6.760E-09 | SLC35F2 | solute carrier family 35, member F2 |
| 5.0 | 1.81E-15 | 1.730E-13 | AMN | amnion associated transmembrane protein |
| 5.0 | 1.58E-11 | 4.510E-10 | ANXA13 | annexin A13 |
| 5.0 | 1.18E-13 | 6.410E-12 | LRAT | lecithin retinol acyltransferase (phosphatidylcholine--retinol O-acyltransferase) |
| 5.0 | 2.00E-08 | 2.300E-07 | CD36 | CD36 molecule (thrombospondin receptor) |
| 4.9 | 4.29E-16 | 4.850E-14 | slc27a5 | Acyl-CoA synthetase involved in bile acid metabolism |
| 4.9 | 7.60E-14 | 4.420E-12 | MAN1A1 | mannosidase, alpha, class 1A, member 1 |
| 4.9 | 1.43E-15 | 1.440E-13 | LPGAT1 | lysophosphatidylglycerol acyltransferase 1 |
| 4.8 | 1.12E-14 | 8.430E-13 | IL15 | interleukin 15 |
| 4.8 | 2.99E-17 | 5.060E-15 | PEPD | peptidase D |
| 4.8 | 8.34E-11 | 1.920E-09 | GZMA | granzyme A (granzyme 1, cytotoxic T-lymphocyte-associated serine esterase 3) |
| 4.7 | 1.95E-09 | 3.000E-08 | FAXDC2 | fatty acid hydroxylase domain containing 2 |
| 4.7 | 2.01E-16 | 2.570E-14 | FER1L6 | fer-1-like 6 (C. elegans) |
| 4.7 | 3.64E-15 | 3.220E-13 | GCH1 | GTP cyclohydrolase 1 |
| 4.6 | 5.68E-16 | 6.300E-14 | MOGAT2 | 2-acylglycerol O-acyltransferase 2-like |
| 4.6 | 1.30E-20 | 7.020E-18 | BAIAP2L2 | BAI1-associated protein 2-like 2 |
| 4.6 | 1.97E-12 | 7.300E-11 | RASGRF2 | Ras protein-specific guanine nucleotide-releasing factor 2 |
| 4.6 | 4.32E-15 | 3.770E-13 | FAAH | fatty acid amide hydrolase |
| 4.6 | 6.61E-11 | 1.580E-09 | SLC5A8 | solute carrier family 5 (sodium/monocarboxylate cotransporter), member 8 |
| 4.5 | 4.32E-12 | 1.480E-10 | PRKG2 | protein kinase, cGMP-dependent, type II |
| 4.5 | 2.41E-08 | 2.700E-07 | IRG1L | immunoresponsive 1 homolog (mouse)-like |
| 4.5 | 2.16E-10 | 4.410E-09 | HSD3B7 | hydroxy-delta-5-steroid dehydrogenase, 3 beta- and steroid delta-isomerase 7 |
| 4.4 | 1.30E-11 | 3.810E-10 | MLN | motilin |
| 4.4 | 6.94E-17 | 1.030E-14 | MAMDC4 | MAM domain containing 4 |
| 4.4 | 1.03E-14 | 7.910E-13 | LRRC58 | leucine rich repeat containing 58 |
| 4.4 | 5.21E-11 | 1.280E-09 | PIK3C2G | phosphatidylinositol-4-phosphate 3-kinase, catalytic subunit type 2 gamma |
| 4.4 | 8.95E-15 | 7.040E-13 | MAP3K15 | mitogen-activated protein kinase kinase kinase 15 |
| 4.4 | 3.84E-17 | 6.270E-15 | ACOT2L | acyl-coenzyme A thioesterase 2, mitochondrial-like |
| 4.3 | 4.84E-15 | 4.160E-13 | DNM1 | dynamin 1 |
| 4.3 | 5.35E-20 | 1.960E-17 | BAAT | bile acid CoA: amino acid N-acyltransferase (glycine N-choloyltransferase) |
| 4.3 | 6.15E-06 | 3.460E-05 | DSEL | dermatan sulfate epimerase-like |
| 4.3 | 1.70E-13 | 8.750E-12 | ANPEP | aminopeptidase N |
| 4.3 | 1.01E-12 | 4.100E-11 | MLXIPL | MLX interacting protein-like |
| 4.2 | 9.84E-09 | 1.250E-07 | CES1 | carboxylesterase 1 (monocyte/macrophage serine esterase 1) |
| 4.2 | 3.11E-08 | 3.390E-07 | SLC10A2 | solute carrier family 10 (sodium/bile acid cotransporter), member 2 |
| 4.2 | 9.79E-13 | 3.990E-11 | SLC6A4 | solute carrier family 6 (neurotransmitter transporter), member 4 |
| 4.1 | 7.32E-12 | 2.310E-10 | MUC2 | mucin 2 |
| 4.1 | 6.69E-17 | 1.010E-14 | NAPEPLD | N-acyl phosphatidylethanolamine phospholipase D |
| 4.1 | 2.84E-06 | 1.750E-05 | TMIGD1 | transmembrane and immunoglobulin domain containing 1 |
| 4.1 | 1.79E-13 | 9.130E-12 | FABP5 | fatty acid binding protein 5 (psoriasis-associated) |
| 4.1 | 6.26E-14 | 3.800E-12 | KBTBD11 | kelch repeat and BTB (POZ) domain containing 11 |
| 4.1 | 9.00E-17 | 1.280E-14 | DDC | dopa decarboxylase (aromatic L-amino acid decarboxylase) |
| 4.1 | 3.48E-11 | 9.020E-10 | IAPP | islet amyloid polypeptide |
| 4.0 | 6.25E-17 | 9.530E-15 | SMPD3 | sphingomyelin phosphodiesterase 3, neutral membrane (neutral sphingomyelinase II) |
| 3.9 | 7.24E-16 | 7.790E-14 | PCSK1 | proprotein convertase subtilisin/kexin type 1 |
| 3.9 | 1.34E-11 | 3.910E-10 | PTPRR | protein tyrosine phosphatase, receptor type, R |
| 3.9 | 1.58E-13 | 8.170E-12 | SLC16A10 | solute carrier family 16 (aromatic amino acid transporter), member 10 |
| 3.9 | 8.82E-10 | 1.500E-08 | GSTT1 | glutathione S-transferase theta 1 |
| 3.8 | 2.48E-13 | 1.220E-11 | KYNU | kynureninase |
| 3.8 | 6.21E-12 | 2.000E-10 | SDR16C5 | short chain dehydrogenase/reductase family 16C, member 5 |
| 3.8 | 1.13E-12 | 4.500E-11 | ACSL5 | acyl-CoA synthetase long-chain family member 5 |
| 3.8 | 9.40E-14 | 5.250E-12 | SH3BP2 | SH3-domain binding protein 2 |
| 3.8 | 2.61E-14 | 1.780E-12 | WWP1 | WW domain containing E3 ubiquitin protein ligase 1 |
| 3.7 | 1.23E-10 | 2.730E-09 | NRG4 | neuregulin 4 |
| 3.7 | 6.17E-12 | 1.990E-10 | BST1 | bone marrow stromal cell antigen 1 |
| 3.7 | 4.44E-10 | 8.250E-09 | MACROD2 | MACRO domain containing 2 |
| 3.7 | 2.01E-10 | 4.140E-09 | TINAG | tubulointerstitial nephritis antigen |
| 3.7 | 7.11E-15 | 5.810E-13 | NR1I3 | nuclear Receptor Subfamily 1 Group I Member 3 |
| 3.7 | 9.96E-17 | 1.380E-14 | CHST6 | carbohydrate (N-acetylglucosamine 6-O) sulfotransferase 6 |
| 3.6 | 1.85E-11 | 5.200E-10 | FGF19 | fibroblast growth factor 19 |
| 3.6 | 7.09E-14 | 4.190E-12 | GDPD1 | glycerophosphodiester phosphodiesterase domain containing 1 |
| 3.6 | 1.41E-13 | 7.460E-12 | PRAP1 | proline-rich acidic protein 1 |
| 3.6 | 3.42E-09 | 4.950E-08 | SLC51B | solute carrier family 51, beta subunit |
| 3.6 | 2.83E-06 | 1.740E-05 | RAG2 | recombination activating gene 2 |
| 3.6 | 6.65E-18 | 1.310E-15 | CDR2 | cerebellar degeneration-related protein 2, 62kDa |
| 3.6 | 6.08E-12 | 1.980E-10 | ATP10A | ATPase, class V, type 10A |
| 3.6 | 2.86E-12 | 1.010E-10 | FOLH1 | folate hydrolase (prostate-specific membrane antigen) 1 |
| 3.6 | 6.25E-11 | 1.500E-09 | FLRT3 | fibronectin leucine rich transmembrane protein 3 |
| 3.6 | 1.17E-12 | 4.680E-11 | NPAS2 | neuronal PAS domain protein 2 |
| 3.5 | 4.76E-11 | 1.180E-09 | EGLN3 | egl-9 family hypoxia-inducible factor 3 |
| 3.5 | 6.65E-14 | 3.980E-12 | DENND5B | DENN/MADD domain containing 5B |
| 3.5 | 3.95E-16 | 4.580E-14 | TRAK1 | trafficking protein, kinesin binding 1 |
| 3.5 | 3.97E-06 | 2.350E-05 | GLP2R | glucagon-like peptide 2 receptor |
| 3.5 | 2.83E-12 | 1.000E-10 | CNDP2 | CNDP dipeptidase 2 (metallopeptidase M20 family) |
| 3.4 | 4.71E-12 | 1.590E-10 | LAMB3 | laminin, beta 3 |
| 3.4 | 1.13E-12 | 4.500E-11 | ISOC1 | isochorismatase domain containing 1 |
| 3.4 | 6.80E-08 | 6.720E-07 | MSMO1 | methylsterol monooxygenase 1 |
| 3.3 | 8.57E-09 | 1.100E-07 | INSIG1 | insulin induced gene 1 |
| 3.3 | 1.56E-12 | 5.920E-11 | XDH | xanthine dehydrogenase |
| 3.3 | 7.77E-16 | 8.230E-14 | DMB1 | MHC class II M beta chain 1 |
| 3.3 | 6.54E-16 | 7.080E-14 | XCL1 | lymphotactin |
| 3.3 | 2.67E-16 | 3.310E-14 | COL17A1 | collagen, type XVII, alpha 1 |
| 3.3 | 1.00E-04 | 5.000E-04 | LPL | lipoprotein lipase |
| 3.3 | 1.55E-11 | 4.430E-10 | GZMK | granzyme K (granzyme 3; tryptase II) |
| 3.3 | 1.13E-13 | 6.200E-12 | AUTS2 | autism susceptibility candidate 2 |
| 3.3 | 5.97E-09 | 8.030E-08 | LIPI | lipase, member I |
| 3.3 | 5.55E-11 | 1.350E-09 | GPR64 | G protein-coupled receptor 64 |
| 3.3 | 1.16E-11 | 3.430E-10 | MAP3K7CL | MAP3K7 C-terminal like |
| 3.3 | 9.87E-11 | 2.230E-09 | TMC7 | transmembrane channel-like 7 |
| 3.3 | 1.13E-10 | 2.520E-09 | TMEM243 | transmembrane protein 243, mitochondrial |
| 3.2 | 2.20E-12 | 8.000E-11 | CRTAM | cytotoxic and regulatory T cell molecule |
| 3.2 | 6.34E-13 | 2.730E-11 | PANX1 | pannexin 1 |
| 3.2 | 5.66E-12 | 1.850E-10 | SELENOP | selenoprotein P, plasma, 1 |
| 3.2 | 2.55E-10 | 5.090E-09 | PDZK1IP1 | PDZK1 interacting protein 1 |
| 3.2 | 1.32E-12 | 5.190E-11 | CROT | carnitine O-octanoyltransferase |
| 3.2 | 2.56E-09 | 3.840E-08 | DAO | D-amino acid oxidase |
| 3.2 | 5.45E-15 | 4.600E-13 | CAB39L | calcium binding protein 39-like |
| 3.1 | 1.56E-14 | 1.130E-12 | BTBD11 | BTB (POZ) domain containing 11 |
| 3.1 | 8.99E-13 | 3.710E-11 | SLCO4A1 | solute carrier organic anion transporter family, member 4A1 |
| 3.1 | 2.85E-10 | 5.610E-09 | C7H2ORF66 | chromosome 7 open reading frame, human C2orf66 |
| 3.1 | 5.82E-08 | 5.830E-07 | CYP2U1 | cytochrome P450 Family 2 Subfamily U Member 1 |
| 3.1 | 2.72E-15 | 2.520E-13 | KIAA0319L | KIAA0319-like |
| 3.1 | 5.85E-13 | 2.550E-11 | FAM8A1 | family with sequence similarity 8, member A1 |
| 3.1 | 4.00E-12 | 1.370E-10 | HNF4G | hepatocyte nuclear factor 4, gamma |
| 3.1 | 3.16E-11 | 8.300E-10 | FAM83F | family with sequence similarity 83, member F |
| 3.1 | 1.04E-06 | 7.170E-06 | SLC13A2 | solute carrier family 13 (sodium-dependent dicarboxylate transporter), member 2 |
| 3.1 | 1.18E-14 | 8.850E-13 | XpNPEP2 | X-prolyl aminopeptidase 2 |
| 3.1 | 1.19E-11 | 3.510E-10 | CCK | cholecystokinin |
| 3.1 | 1.02E-11 | 3.070E-10 | TM4SF1 | transmembrane 4 L six family member 1 |
| 3.1 | 1.51E-08 | 1.800E-07 | ZP4 | zona pellucida glycoprotein 4 |
| 3.0 | 6.84E-12 | 2.180E-10 | HS3ST1 | heparan sulfate (glucosamine) 3-O-sulfotransferase 1 |
| 3.0 | 9.50E-13 | 3.890E-11 | VAT1 | vesicle amine transport 1 |
| 3.0 | 3.76E-12 | 1.300E-10 | ZFPM1 | zinc finger protein, FOG family member 1 |
| 3.0 | 5.76E-11 | 1.390E-09 | SLC18B1 | solute carrier family 18, subfamily B, member 1 |
| 3.0 | 8.97E-13 | 3.710E-11 | ABCC10 | ATP-binding cassette, sub-family C (CFTR/MRP), member 10 |
| 3.0 | 4.57E-11 | 1.140E-09 | FGF9 | fibroblast growth factor 9 (glia-activating factor) |
| 3.0 | 7.03E-13 | 3.000E-11 | PGAP1 | post-GPI attachment to proteins 1 |
| 3.0 | 2.03E-13 | 1.020E-11 | ABCD2 | ATP-binding cassette, sub-family D (ALD), member 2 |
| 3.0 | 2.26E-13 | 1.120E-11 | FRMD1 | FERM domain containing 1 |
| 3.0 | 1.40E-12 | 5.430E-11 | MAOA | monoamine oxidase A |
| 3.0 | 1.09E-13 | 6.010E-12 | OSBPL1A | oxysterol binding protein-like 1A |
| 3.0 | 9.44E-10 | 1.590E-08 | TKFC | triokinase and FMN cyclase |
| 2.9 | 2.33E-13 | 1.150E-11 | MAP3K4 | mitogen-activated protein kinase kinase kinase 4 |
| 2.9 | 9.10E-13 | 3.750E-11 | NEDD9 | neural precursor cell expressed, developmentally down-regulated 9 |
| 2.9 | 8.80E-15 | 6.940E-13 | MYRF | myelin regulatory factor |
| 2.9 | 7.75E-13 | 3.250E-11 | SCP2 | sterol carrier protein 2 |
| 2.9 | 2.39E-11 | 6.510E-10 | EMB | embigin |
| 2.9 | 4.70E-13 | 2.120E-11 | DAPK1 | death-associated protein kinase 1 isoform 1 |
| 2.9 | 4.14E-11 | 1.040E-09 | CIITA | class II, major histocompatibility complex, transactivator-like |
| 2.9 | 1.53E-13 | 7.960E-12 | STAP1 | signal transducing adaptor family member 1 |
| 2.9 | 8.83E-10 | 1.500E-08 | KL | klotho |
| 2.9 | 1.21E-13 | 6.570E-12 | IFFO2 | intermediate filament family orphan 2 |
| 2.9 | 1.23E-12 | 4.830E-11 | KY | kyphoscoliosis peptidase |
| 2.9 | 1.52E-11 | 4.370E-10 | MCF2 | MCF.2 cell line derived transforming sequence |
| 2.9 | 6.14E-14 | 3.760E-12 | SLC27A4 | solute carrier family 27 (fatty acid transporter), member 4 |
| 2.8 | 1.21E-14 | 8.880E-13 | CD226 | CD226 molecule |
| 2.8 | 2.13E-13 | 1.070E-11 | OSR2 | odd-skipped related transciption factor 2 |
| 2.8 | 2.22E-09 | 3.380E-08 | ATP7B | ATPase, Cu++ transporting, beta polypeptide |
| 2.8 | 1.09E-08 | 1.360E-07 | SLC6A14 | solute carrier family 6 (amino acid transporter), member 14 |
| 2.8 | 5.14E-15 | 4.350E-13 | GNAL | guanine nucleotide binding protein (G protein), alpha activating activity polypeptide, olfactory type |
| 2.8 | 1.03E-12 | 4.150E-11 | VIPR1 | vasoactive intestinal peptide receptor 1 |
| 2.8 | 7.83E-10 | 1.360E-08 | EFCAB4B | EF-hand calcium binding domain 4B |
| 2.8 | 2.59E-15 | 2.410E-13 | MGAT3 | mannosyl (beta-1,4-)-glycoprotein beta-1,4-N-acetylglucosaminyltransferase |
| 2.8 | 2.87E-07 | 2.350E-06 | PDK4 | pyruvate dehydrogenase kinase, isozyme 4 |
| 2.8 | 6.70E-14 | 3.990E-12 | RHPN1 | rhophilin, Rho GTPase binding protein 1 |
| 2.8 | 1.44E-09 | 2.310E-08 | ROS1 | ROS proto-oncogene 1 , receptor tyrosine kinase |
| 2.8 | 1.27E-13 | 6.820E-12 | AHCYL2 | adenosylhomocysteinase-like 2 |
| 2.8 | 1.44E-09 | 2.310E-08 | AIFM3 | apoptosis inducing factor, mitochondria associated 3 |
| 2.8 | 6.84E-11 | 1.620E-09 | EPT1 | ethanolaminephosphotransferase 1 (CDP-ethanolamine-specific) |
| 2.8 | 2.58E-09 | 3.860E-08 | SFXN1 | sideroflexin 1 |
| 2.7 | 3.98E-12 | 1.370E-10 | OSBPL6 | oxysterol binding protein-like 6 |
| 2.7 | 5.61E-13 | 2.470E-11 | SOAT1 | sterol O-acyltransferase 1 |
| 2.7 | 9.80E-06 | 5.260E-05 | TRPM6 | transient receptor potential cation channel, subfamily M, member 6 |
| 2.7 | 4.13E-10 | 7.750E-09 | CAPN13 | calpain 13 |
| 2.7 | 1.84E-10 | 3.840E-09 | CENPV | centromere protein V |
| 2.7 | 1.54E-13 | 8.000E-12 | TMPRSS7 | transmembrane protease, serine 7 |
| 2.7 | 4.32E-10 | 8.050E-09 | CD7 | CD7 molecule |
| 2.7 | 2.17E-13 | 1.080E-11 | SGPL1 | sphingosine-1-phosphate lyase 1 |
| 2.7 | 2.46E-10 | 4.930E-09 | TCRD | T cell receptor delta chain |
| 2.7 | 5.22E-08 | 5.310E-07 | MAT2A | S-adenosylmethionine synthase isoform type-2-like |
| 2.7 | 1.10E-11 | 3.270E-10 | FLVCR2 | feline leukemia virus subgroup C cellular receptor family, member 2 |
| 2.7 | 3.27E-09 | 4.750E-08 | GK | glycerol kinase |
| 2.7 | 2.47E-13 | 1.220E-11 | LYN | v-yes-1 Yamaguchi sarcoma viral related oncogene homolog |
| 2.7 | 9.66E-10 | 1.630E-08 | SLC23A1 | Solute Carrier Family 23 Member 1 |
| 2.7 | 5.71E-14 | 3.520E-12 | AGPAT3 | 1-acylglycerol-3-phosphate O-acyltransferase 3 |
| 2.7 | 3.08E-15 | 2.790E-13 | ATRN | Attractin |
| 2.7 | 5.33E-10 | 9.640E-09 | FABP1 | fatty acid binding protein 1, liver |
| 2.7 | 8.51E-15 | 6.740E-13 | ITK | IL2-inducible T-cell kinase |
| 2.7 | 2.00E-09 | 3.080E-08 | GPAM | glycerol-3-phosphate acyltransferase, mitochondrial |
| 2.7 | 2.53E-12 | 9.030E-11 | GPR126 | G protein-coupled receptor 126 |
| 2.7 | 2.51E-10 | 5.000E-09 | RYR3 | ryanodine receptor 3 |
| 2.7 | 6.02E-12 | 1.960E-10 | TDP2 | tyrosyl-DNA phosphodiesterase 2 |
| 2.7 | 5.36E-14 | 3.360E-12 | VIL1 | villin 1 |
| 2.7 | 9.13E-10 | 1.550E-08 | C5H14ORF159 | chromosome 5 open reading frame, human C14orf159 |
| 2.7 | 7.68E-14 | 4.420E-12 | DOCK9 | dedicator of cytokinesis 9 |
| 2.7 | 8.69E-10 | 1.490E-08 | TMEM135 | transmembrane protein 135 |
| 2.7 | 9.89E-10 | 1.660E-08 | KIAA1211 | KIAA1211 |
| 2.7 | 4.07E-08 | 4.270E-07 | FALG | Fas ligand |
| 2.7 | 3.53E-12 | 1.230E-10 | PISD | phosphatidylserine decarboxylase |
| 2.6 | 3.46E-13 | 1.630E-11 | CCL1 | chemokine (C-C motif) ligand 1 |
| 2.6 | 1.67E-09 | 2.620E-08 | TMEM37 | transmembrane protein 37 |
| 2.6 | 2.75E-13 | 1.330E-11 | TJAP1 | tight junction associated protein 1 (peripheral) |
| 2.6 | 5.89E-15 | 4.950E-13 | MTSS1 | metastasis suppressor 1 |
| 2.6 | 7.85E-09 | 1.020E-07 | PRMT8 | protein arginine methyltransferase 8 |
| 2.6 | 2.37E-10 | 4.780E-09 | FBXO8 | F-box protein 8 |
| 2.6 | 1.69E-10 | 3.580E-09 | HADH | hydroxyacyl-CoA dehydrogenase |
| 2.6 | 5.17E-11 | 1.270E-09 | CD8A | CD8a molecule |
| 2.6 | 1.42E-12 | 5.470E-11 | GNE | glucosamine (UDP-N-acetyl)-2-epimerase/N-acetylmannosamine kinase |
| 2.6 | 1.02E-11 | 3.070E-10 | LZTFL1 | leucine zipper transcription factor-like 1 |
| 2.6 | 2.39E-11 | 6.510E-10 | CYP4B1L | cytochrome P450 4B1-like |
| 2.6 | 2.30E-10 | 4.650E-09 | CD200R1L | CD200 receptor 1-like |
| 2.6 | 1.30E-11 | 3.820E-10 | SLC25A16 | solute carrier family 25 (mitochondrial carrier), member 16 |
| 2.6 | 6.79E-11 | 1.610E-09 | AQP5 | Aquaporin 5 |
| 2.6 | 1.96E-10 | 4.040E-09 | IL12RB2 | interleukin 12 receptor, beta 2 |
| 2.5 | 2.73E-11 | 7.290E-10 | AADACL2 | arylacetamide deacetylase-like 2 |
| 2.5 | 3.16E-10 | 6.160E-09 | GUCY2C | guanylate cyclase 2C |
| 2.5 | 1.94E-12 | 7.210E-11 | PARP8 | poly (ADP-ribose) polymerase family, member 8 |
| 2.5 | 6.20E-09 | 8.310E-08 | PER2 | period circadian clock 2 |
| 2.5 | 1.32E-12 | 5.190E-11 | ACSL4 | acyl-CoA synthetase long-chain family member 4 |
| 2.5 | 8.19E-12 | 2.540E-10 | ARHGAP10 | Rho GTPase activating protein 10 |
| 2.5 | 1.00E-12 | 4.070E-11 | C8H1ORF168 | chromosome 8 open reading frame, human C1orf168 |
| 2.5 | 5.67E-05 | 2.000E-04 | FBLN5 | fibulin 5 |
| 2.5 | 8.40E-13 | 3.520E-11 | TOX | thymocyte selection-associated high mobility group box |
| 2.5 | 5.60E-14 | 3.480E-12 | TOM1L2 | target of myb1-like 2 |
| 2.5 | 2.75E-09 | 4.060E-08 | RALGPS1 | Ral GEF with PH domain and SH3 binding motif 1 |
| 2.5 | 4.02E-11 | 1.020E-09 | agmat | agmatinase, mitochondrial precursor |
| 2.5 | 2.39E-11 | 6.510E-10 | GPR55 | G protein-coupled receptor 55 |
| 2.5 | 4.56E-09 | 6.320E-08 | SAT1 | spermidine/spermine N1-acetyltransferase 1 |
| 2.5 | 8.01E-14 | 4.580E-12 | TESK2 | testis-specific kinase 2 |
| 2.5 | 3.97E-09 | 5.620E-08 | EHHADH | enoyl-CoA, hydratase/3-hydroxyacyl CoA dehydrogenase |
| 2.5 | 5.78E-14 | 3.550E-12 | ACOT11 | acyl-CoA thioesterase 11 |
| 2.5 | 8.42E-12 | 2.590E-10 | ADAMTS17 | ADAM metallopeptidase with thrombospondin type 1 motif, 17 |
| 2.5 | 7.31E-12 | 2.310E-10 | IGSF5 | immunoglobulin superfamily, member 5 |
| 2.5 | 1.43E-12 | 5.490E-11 | CORO2A | coronin, actin binding protein, 2A |
| 2.5 | 3.31E-07 | 2.650E-06 | MYOM2 | myomesin 2 |
| 2.5 | 7.87E-12 | 2.460E-10 | PPP1R16B | protein phosphatase 1, regulatory subunit 16B |
| 2.5 | 2.97E-08 | 3.250E-07 | RASD1 | RAS, dexamethasone-induced 1 |
| 2.5 | 1.63E-07 | 1.440E-06 | PPARA | peroxisome proliferator-activated receptor alpha |
| 2.5 | 9.15E-13 | 3.760E-11 | PRKCH | protein kinase C eta |
| 2.5 | 6.78E-13 | 2.910E-11 | PROSER2 | proline and serine rich 2 |
| 2.5 | 8.06E-11 | 1.860E-09 | RUFY2 | RUN and FYVE domain containing 2 |
| 2.5 | 4.01E-09 | 5.670E-08 | SEC22C | SEC22 homolog C, vesicle trafficking protein |
| 2.5 | 5.94E-11 | 1.430E-09 | SLC7A6 | solute carrier family 7 (amino acid transporter light chain, y+L system), member 6 |
| 2.4 | 4.91E-11 | 1.220E-09 | CCSER2 | coiled-coil serine-rich protein 2 |
| 2.4 | 2.15E-06 | 1.370E-05 | DNAH14 | dynein, axonemal, heavy chain 14 |
| 2.4 | 1.70E-07 | 1.490E-06 | ATP2B2 | ATPase, Ca++ transporting, plasma membrane 2 |
| 2.4 | 1.49E-08 | 1.790E-07 | CIDEA | cell death-inducing DFFA-like effector a |
| 2.4 | 7.37E-10 | 1.290E-08 | F11 | coagulation factor XI |
| 2.4 | 2.94E-06 | 1.800E-05 | C8ORF22 | chromosome 2 open reading frame, human C8orf22 |
| 2.4 | 2.79E-08 | 3.080E-07 | KCNK5 | potassium channel, two pore domain subfamily K, member 5 |
| 2.4 | 4.47E-12 | 1.520E-10 | NUAK2 | NUAK family, SNF1-like kinase, 2 |
| 2.4 | 2.56E-09 | 3.840E-08 | OIT3 | oncoprotein induced transcript 3 |
| 2.4 | 6.76E-10 | 1.200E-08 | SLC16A6 | solute carrier family 16, member 6 |
| 2.4 | 6.44E-13 | 2.770E-11 | SST | Somatostatin Somatostatin-28 Somatostatin-14 |
| 2.4 | 1.82E-13 | 9.280E-12 | EXOC3L4 | exocyst complex component 3-like 4 |
| 2.4 | 8.63E-09 | 1.110E-07 | PFKFB3 | 6-phosphofructo-2-kinase/fructose-2,6-biphosphatase 3 |
| 2.4 | 7.14E-13 | 3.040E-11 | PNPLA2 | patatin-like phospholipase domain containing 2 |
| 2.4 | 8.21E-10 | 1.420E-08 | SOSTDC1 | sclerostin domain containing 1 |
| 2.4 | 2.63E-09 | 3.910E-08 | FOXP2 | forkhead box P2 |
| 2.4 | 3.74E-10 | 7.110E-09 | SLC4A7 | solute carrier family 4, sodium bicarbonate cotransporter, member 7 |
| 2.4 | 1.64E-10 | 3.490E-09 | GRHPR | glyoxylate reductase/hydroxypyruvate reductase |
| 2.4 | 2.19E-09 | 3.350E-08 | HERC3 | hect domain and RLD 3 |
| 2.4 | 1.35E-13 | 7.210E-12 | JAKMIP1 | janus kinase and microtubule interacting protein 1 |
| 2.4 | 2.32E-11 | 6.380E-10 | ARHGAP36 | Rho GTPase activating protein 36 |
| 2.4 | 1.69E-08 | 1.990E-07 | BG1 | BG-like antigen 1 |
| 2.4 | 5.52E-12 | 1.820E-10 | C4H4orf50 | chromosome 4 open reading frame, human C4orf50 |
| 2.4 | 4.11E-10 | 7.710E-09 | CCBE1 | collagen and calcium binding EGF domains 1 |
| 2.4 | 2.02E-12 | 7.430E-11 | RIC8B | RIC8 guanine nucleotide exchange factor B |
| 2.4 | 2.30E-06 | 1.450E-05 | SLC30A10 | solute carrier family 30, member 10 |
| 2.4 | 2.48E-10 | 4.960E-09 | SPG20 | spastic paraplegia 20 (Troyer syndrome) |
| 2.4 | 6.79E-11 | 1.610E-09 | SYTL5 | synaptotagmin-like 5 |
| 2.4 | 2.85E-11 | 7.550E-10 | DHRS11 | dehydrogenase/reductase (SDR family) member 11 |
| 2.4 | 5.45E-08 | 5.510E-07 | F7 | coagulation factor VII (serum prothrombin conversion accelerator) |
| 2.4 | 3.89E-09 | 5.520E-08 | FRMD4B | FERM domain containing 4B |
| 2.4 | 5.17E-14 | 3.280E-12 | MXI1 | MAX interactor 1, dimerization protein |
| 2.4 | 6.09E-13 | 2.640E-11 | TEC | tec protein tyrosine kinase |
| 2.3 | 7.86E-08 | 7.610E-07 | ATP8B1 | ATPase, class I, type 8B, member 1 |
| 2.3 | 8.01E-11 | 1.850E-09 | PTBP3 | polypyrimidine tract binding protein 3 |
| 2.3 | 3.74E-07 | 2.940E-06 | GPR112 | G protein-coupled receptor 112 |
| 2.3 | 1.21E-12 | 4.830E-11 | FAM13A | family with sequence similarity 13, member A |
| 2.3 | 5.47E-10 | 9.870E-09 | ZNF502 | zinc finger protein 502 |
| 2.3 | 5.82E-11 | 1.410E-09 | ABHD6 | abhydrolase domain containing 6 |
| 2.3 | 3.87E-13 | 1.790E-11 | IL2RB | interleukin-2 receptor subunit beta-like |
| 2.3 | 1.13E-12 | 4.500E-11 | TBX3 | T-box 3 |
| 2.3 | 8.18E-09 | 1.060E-07 | ABCG2 | ATP-binding cassette, sub-family G (WHITE), member 2 (Junior blood group) |
| 2.3 | 5.58E-12 | 1.840E-10 | PTPN22 | protein tyrosine phosphatase, non-receptor type 22 (lymphoid) |
| 2.3 | 3.49E-13 | 1.650E-11 | SLC30A1 | solute carrier family 30 (zinc transporter), member 1 |
| 2.3 | 2.12E-09 | 3.250E-08 | DUSP16 | dual specificity phosphatase 16 |
| 2.3 | 7.91E-10 | 1.380E-08 | IL18 | interleukin 18 |
| 2.3 | 5.85E-08 | 5.850E-07 | SOT3A1L | sulfotransferase family 3A, member 1-like |
| 2.3 | 9.15E-13 | 3.760E-11 | PELI2 | pellino homolog 2 (Drosophila) |
| 2.3 | 2.71E-11 | 7.270E-10 | RAB40B | RAB40B, member RAS oncogene family |
| 2.3 | 6.80E-11 | 1.610E-09 | SLC37A4 | solute carrier family 37 (glucose-6-phosphate transporter), member 4 |
| 2.3 | 4.00E-04 | 1.300E-03 | KIRREL3 | kirre like nephrin family adhesion molecule 3 |
| 2.3 | 2.25E-10 | 4.580E-09 | LCORL | ligand dependent nuclear receptor corepressor-like |
| 2.3 | 1.87E-10 | 3.900E-09 | PAFAH2 | platelet activating factor acetylhydrolase 2 |
| 2.3 | 1.72E-09 | 2.710E-08 | TMEM231 | Transmembrane Protein 231 |
| 2.3 | 4.89E-08 | 5.020E-07 | ART1 | ADP-ribosyltransferase 1 |
| 2.3 | 5.49E-11 | 1.340E-09 | CNDP1 | carnosine dipeptidase 1 (metallopeptidase M20 family) |
| 2.3 | 3.63E-06 | 2.170E-05 | MAB21L2 | mab-21-like 2 (C. elegans) |
| 2.3 | 2.43E-08 | 2.720E-07 | PHYH | phytanoyl-CoA 2-hydroxylase |
| 2.3 | 7.27E-08 | 7.130E-07 | CCDC147 | coiled-coil domain containing 147 |
| 2.3 | 4.14E-09 | 5.810E-08 | RASGRP1 | RAS guanyl releasing protein 1 (calcium and DAG-regulated) |
| 2.3 | 2.49E-07 | 2.070E-06 | SIK1 | salt-inducible kinase 1 |
| 2.2 | 2.30E-14 | 1.590E-12 | INPP5B | inositol polyphosphate-5-phosphatase, 75kDa |
| 2.2 | 1.98E-07 | 1.710E-06 | TGM4 | transglutaminase 4 (prostate) |
| 2.2 | 3.85E-09 | 5.470E-08 | BCHE | butyrylcholinesterase |
| 2.2 | 2.74E-05 | 1.000E-04 | CSTA | cystatin A (stefin A) |
| 2.2 | 6.78E-12 | 2.170E-10 | NRIP1 | nuclear receptor interacting protein 1 |
| 2.2 | 4.10E-10 | 7.700E-09 | PECR | peroxisomal trans-2-enoyl-CoA reductase |
| 2.2 | 2.85E-10 | 5.610E-09 | RIPK3 | receptor-interacting serine-threonine kinase 3 |
| 2.2 | 2.11E-07 | 1.790E-06 | AGPAT9 | 1-acylglycerol-3-phosphate O-acyltransferase 9 |
| 2.2 | 1.10E-07 | 1.020E-06 | ATOH7 | atonal bHLH transcription factor 7 |
| 2.2 | 3.10E-13 | 1.480E-11 | OTUD7A | OTU domain containing 7A |
| 2.2 | 1.44E-08 | 1.740E-07 | RALY | RALY heterogeneous nuclear ribonucleoprotein |
| 2.2 | 3.94E-10 | 7.470E-09 | SH3BP1 | SH3-domain binding protein 1 |
| 2.2 | 6.23E-12 | 2.000E-10 | SLC25A22 | solute carrier family 25 (mitochondrial carrier: glutamate), member 22 |
| 2.2 | 6.19E-11 | 1.490E-09 | TMEM181 | transmembrane protein 181 |
| 2.2 | 2.48E-11 | 6.730E-10 | ABHD2 | abhydrolase domain containing 2 |
| 2.2 | 6.76E-07 | 4.930E-06 | CH17-360D5.1 | neuropeptide Y receptor Y4 |
| 2.2 | 7.26E-12 | 2.300E-10 | CLCN6 | chloride channel, voltage-sensitive 6 |
| 2.2 | 2.36E-11 | 6.450E-10 | DISP1 | dispatched homolog 1 (Drosophila) |
| 2.2 | 7.55E-09 | 9.840E-08 | GPR180 | G protein-coupled receptor 180 |
| 2.2 | 4.94E-09 | 6.790E-08 | NPY6R | neuropeptide Y receptor Y6 |
| 2.2 | 2.52E-12 | 9.010E-11 | RNPEP | arginyl aminopeptidase (aminopeptidase B) |
| 2.2 | 7.64E-13 | 3.220E-11 | CERS1 | ceramide synthase 1 |
| 2.2 | 5.38E-07 | 4.030E-06 | KBP | kainate binding protein |
| 2.2 | 4.32E-06 | 2.540E-05 | LAPTM4B | lysosomal protein transmembrane 4 beta |
| 2.2 | 1.12E-08 | 1.390E-07 | WWC2 | WW and C2 domain containing 2 |
| 2.2 | 3.56E-09 | 5.140E-08 | CAPN8 | calpain 8 |
| 2.2 | 2.95E-10 | 5.790E-09 | DAW1 | dynein assembly factor with WDR repeat domains 1 |
| 2.2 | 4.79E-10 | 8.800E-09 | GKAP1 | G kinase anchoring protein 1 |
| 2.2 | 5.55E-08 | 5.610E-07 | GPCPD1 | glycerophosphocholine phosphodiesterase GDE1 homolog (S. cerevisiae) |
| 2.2 | 5.83E-11 | 1.410E-09 | IKZF2 | IKAROS family zinc finger 2 (Helios) |
| 2.2 | 4.81E-12 | 1.610E-10 | LGALS2 | lectin, galactoside-binding, soluble, 2 |
| 2.2 | 1.71E-10 | 3.610E-09 | MCU | mitochondrial calcium uniporter |
| 2.2 | 9.97E-10 | 1.670E-08 | retreg1 | reticulophagy regulator 1 |
| 2.2 | 1.98E-10 | 4.090E-09 | TRAT1 | T cell receptor associated transmembrane adaptor 1 |
| 2.2 | 1.44E-10 | 3.130E-09 | CYP2J2L3 | cytochrome P450, family 2, subfamily J, polypeptide 2-like 3 |
| 2.2 | 3.61E-09 | 5.210E-08 | MCUR1 | mitochondrial calcium uniporter regulator 1 |
| 2.2 | 1.62E-07 | 1.440E-06 | PDCD2L | programmed cell death 2-like |
| 2.2 | 1.51E-12 | 5.770E-11 | SYTL3 | synaptotagmin-like 3 |
| 2.2 | 4.76E-12 | 1.600E-10 | TSHZ1 | teashirt zinc finger homeobox 1 |
| 2.2 | 7.76E-10 | 1.350E-08 | ATL2 | atlastin GTPase 2 |
| 2.2 | 3.81E-09 | 5.440E-08 | BMP3 | bone morphogenetic protein 3 |
| 2.2 | 7.30E-08 | 7.160E-07 | F2R | coagulation factor II (thrombin) receptor |
| 2.2 | 1.31E-10 | 2.860E-09 | PXDC1 | PX domain containing 1 |
| 2.1 | 1.59E-10 | 3.410E-09 | DOCK5 | dedicator of cytokinesis 5 |
| 2.1 | 3.72E-11 | 9.520E-10 | H6PD | hexose-6-phosphate dehydrogenase (glucose 1-dehydrogenase) |
| 2.1 | 1.20E-14 | 8.880E-13 | SRGAP3 | SLIT-ROBO Rho GTPase activating protein 3 |
| 2.1 | 1.38E-12 | 5.370E-11 | ACADL | acyl-CoA dehydrogenase, long chain |
| 2.1 | 2.65E-12 | 9.430E-11 | ARHGAP18 | Rho GTPase activating protein 18 |
| 2.1 | 1.93E-12 | 7.170E-11 | ARHGEF18 | Rho/Rac guanine nucleotide exchange factor (GEF) 18 |
| 2.1 | 5.42E-12 | 1.800E-10 | ARHGEF3 | Rho guanine nucleotide exchange factor (GEF) 3 |
| 2.1 | 4.48E-06 | 2.620E-05 | GRID2 | glutamate receptor, ionotropic, delta 2 |
| 2.1 | 2.61E-07 | 2.160E-06 | P2RX1 | purinergic receptor P2X, ligand-gated ion channel, 1 |
| 2.1 | 6.09E-07 | 4.500E-06 | CCL20 | chemokine (C-C motif) ligand 20 |
| 2.1 | 1.25E-10 | 2.760E-09 | COBL | cordon-bleu WH2 repeat protein |
| 2.1 | 1.35E-11 | 3.920E-10 | FGD3 | FYVE, RhoGEF and PH domain containing 3 |
| 2.1 | 2.53E-09 | 3.810E-08 | FNIP1 | folliculin interacting protein 1 |
| 2.1 | 2.66E-10 | 5.270E-09 | IL7R | interleukin 7 receptor |
| 2.1 | 5.55E-10 | 9.980E-09 | SLC35A5 | solute carrier family 35, member A5 |
| 2.1 | 5.47E-08 | 5.530E-07 | FNIP2 | folliculin interacting protein 2 |
| 2.1 | 1.32E-06 | 8.880E-06 | HEY2 | hairy/enhancer-of-split related family bHLH transcription factor with YRPW motif |
| 2.1 | 3.51E-10 | 6.760E-09 | LRRC8B | leucine rich repeat containing 8 family, member B |
| 2.1 | 3.84E-12 | 1.330E-10 | PLIN4 | Perilipin 4 |
| 2.1 | 4.80E-10 | 8.810E-09 | RORA | RAR-related orphan receptor A |
| 2.1 | 1.15E-09 | 1.880E-08 | SAR1B | SAR1 homolog B (S. cerevisiae) |
| 2.1 | 2.00E-04 | 7.000E-04 | snoRNA RF00431 |  |
| 2.1 | 2.76E-09 | 4.080E-08 | ASAP2 | ArfGAP with SH3 domain, ankyrin repeat and PH domain 2 |
| 2.1 | 1.95E-06 | 1.250E-05 | HPGD | hydroxyprostaglandin dehydrogenase 15-(NAD) |
| 2.1 | 4.44E-12 | 1.520E-10 | LRRC16A | leucine rich repeat containing 16A |
| 2.1 | 5.77E-08 | 5.790E-07 | ABCC6 | ATP-binding cassette, sub-family C (CFTR/MRP), member 6 |
| 2.1 | 1.57E-10 | 3.380E-09 | CASP3 | caspase 3, apoptosis-related cysteine peptidase |
| 2.1 | 5.69E-10 | 1.020E-08 | CD3D | CD3d molecule, delta (CD3-TCR complex) |
| 2.1 | 1.39E-12 | 5.410E-11 | MYRIP | myosin VIIA and Rab interacting protein |
| 2.1 | 4.42E-11 | 1.110E-09 | PARK2 | parkin RBR E3 ubiquitin protein ligase |
| 2.1 | 7.83E-11 | 1.810E-09 | TYRO3 | TYRO3 protein tyrosine kinase |
| 2.1 | 3.10E-03 | 8.500E-03 | ALDH1A3 | aldehyde dehydrogenase 1 family, member A3 |
| 2.1 | 1.11E-06 | 7.660E-06 | CCL19 | C-C Motif Chemokine Ligand 19 |
| 2.1 | 1.16E-11 | 3.440E-10 | CLCN5 | chloride channel, voltage-sensitive 5 |
| 2.1 | 4.41E-08 | 4.580E-07 | GPR18 | G protein-coupled receptor 18 |
| 2.1 | 8.78E-08 | 8.390E-07 | PARP14 | poly (ADP-ribose) polymerase family, member 14; poly [ADP-ribose] polymerase 14-like |
| 2.1 | 3.29E-11 | 8.580E-10 | SLC8B1 | solute carrier family 8 (sodium/lithium/calcium exchanger), member B1 |
| 2.1 | 2.95E-08 | 3.240E-07 | CCR9 | chemokine (C-C motif) receptor 9 |
| 2.1 | 4.25E-08 | 4.440E-07 | CDC14A | cell division cycle 14° |
| 2.1 | 2.00E-08 | 2.300E-07 | FASLG | Fas ligand (TNF superfamily, member 6) |
| 2.1 | 1.36E-06 | 9.110E-06 | FLVCR1 | feline leukemia virus subgroup C cellular receptor 1 |
| 2.1 | 5.31E-14 | 3.340E-12 | EPN2 | epsin 2 |
| 2.1 | 1.00E-10 | 2.270E-09 | PPP1R3B | protein phosphatase 1, regulatory subunit 3B |
| 2.1 | 5.24E-09 | 7.150E-08 | RASGEF1C | RasGEF domain family, member 1C |
| 2.1 | 6.57E-15 | 5.390E-13 | ARHGAP26 | Rho GTPase activating protein 26 |
| 2.1 | 3.63E-08 | 3.860E-07 | C10ORF11 | chromosome 6 open reading frame, human C10orf11 |
| 2.1 | 3.49E-10 | 6.740E-09 | C8H1ORF21 | chromosome 8 open reading frame, human C1orf21 |
| 2.1 | 2.67E-11 | 7.180E-10 | CD3E | CD3e molecule, epsilon (CD3-TCR complex) |
| 2.1 | 2.53E-12 | 9.040E-11 | CPNE2 | copine II |
| 2.1 | 6.90E-09 | 9.120E-08 | DOK6 | docking protein 6 |
| 2.1 | 2.86E-06 | 1.760E-05 | FSHR | follicle stimulating hormone receptor |
| 2.1 | 4.94E-07 | 3.750E-06 | HEBP1 | heme binding protein 1 |
| 2.1 | 1.12E-07 | 1.040E-06 | TPPP | tubulin polymerization promoting protein |
| 2.1 | 4.52E-13 | 2.070E-11 | WWC1 | WW and C2 domain containing 1 |
| 2.0 | 9.07E-08 | 8.620E-07 | ARAP2 | ArfGAP with RhoGAP domain, ankyrin repeat and PH domain 2 |
| 2.0 | 1.54E-09 | 2.450E-08 | IQSEC1 | IQ motif and Sec7 domain 1 |
| 2.0 | 1.89E-08 | 2.200E-07 | SLC22A4 | solute carrier family 22 (organic cation transporter), member 4 |
| 2.0 | 1.13E-07 | 1.040E-06 | TXK | TXK tyrosine kinase |
| 2.0 | 9.97E-08 | 9.380E-07 | C26H6orf222 | chromosome 26 open reading frame, human C6orf222 |
| 2.0 | 4.66E-12 | 1.580E-10 | GRAP2 | GRB2-related adaptor protein 2 |
| 2.0 | 8.86E-08 | 8.450E-07 | KLHL38 | kelch-like family member 38 |
| 2.0 | 5.73E-10 | 1.030E-08 | PPIP5K1 | diphosphoinositol pentakisphosphate kinase 1 |
| 2.0 | 6.02E-13 | 2.620E-11 | STK17B | serine/threonine kinase 17b |
| 2.0 | 9.37E-10 | 1.590E-08 | VTCN1L | V-set domain containing T cell activation inhibitor 1-like |
| 2.0 | 3.15E-07 | 2.540E-06 | XCR1 | chemokine (C motif) receptor 1 |
| 2.0 | 5.73E-12 | 1.870E-10 | AMDHD2 | amidohydrolase domain containing 2 |
| 2.0 | 3.83E-08 | 4.050E-07 | CAPN9 | calpain 9 |
| 2.0 | 1.34E-06 | 8.990E-06 | DHCR7 | 7-dehydrocholesterol reductase |
| 2.0 | 1.08E-10 | 2.420E-09 | FAM126A | family with sequence similarity 126, member A |
| 2.0 | 9.49E-11 | 2.160E-09 | PHLPP2 | PH domain and leucine rich repeat protein phosphatase 2 |
| 2.0 | 5.20E-09 | 7.090E-08 | SGK1 | serum/glucocorticoid regulated kinase 1 |
| 2.0 | 3.84E-08 | 4.060E-07 | TMEM41B | transmembrane protein 41B |
| 2.0 | 3.22E-09 | 4.690E-08 | ACSL3 | acyl-CoA synthetase long-chain family member 3 |
| 2.0 | 5.03E-10 | 9.130E-09 | CCL5 | chemokine (C-C motif) ligand 5 |
| 2.0 | 4.29E-11 | 1.080E-09 | SLC1A7 | solute carrier family 1 (glutamate transporter), member 7 |
| 2.0 | 3.64E-07 | 2.870E-06 | FNDC7 | fibronectin type III domain containing 7 |
| 2.0 | 1.75E-05 | 8.780E-05 | NELL2 | NEL-like 2 |
| 2.0 | 5.51E-10 | 9.930E-09 | STS | steroid sulfatase (microsomal), isozyme S |
| 2.0 | 3.72E-10 | 7.080E-09 | TMEM65 | transmembrane protein 65 |

Transcripts obtained with Transcriptomic Analysis Console (TAC) Affymetrix© software (4.0.1.36) were considered as differentially expressed transcripts (DET) when showing a >=2-fold change ratio (FCR)^1^ and a False Discovery Rate (FDR)^2^ < 0.05 between tissues.

^3^ Transcripts were annotated based on *Gallus gallus* Ensembl (release 85, [www.ensembl.org](http://www.ensembl.org)).
