# Supplementary material for: Exploring Differential Transcriptome between Jejunal and Cecal Tissue of Broiler Chickens
Source: Animals (Basel). 2019 May 7;9(5):221. doi: 10.3390/ani9050221 (PMC6562892; doi:10.3390/ani9050221)
Supplement: Supplementary file 1 [file animals-09-00221-s001.zip › supplementary files/Table S 3.docx]

**Supplementary Table 3**. List of the assigned differentially expressed transcripts in cecal mucosa of broiler chickens ranked for the fold change ratio (FCR), compared to jejunal mucosa.

| Fold Change^1^ | *P*-value | FDR  *P*-value^2^ | Gene  Symbol^3^ | Description |
| --- | --- | --- | --- | --- |
| 244.4 | 6.170E-21 | 4.18E-18 | CBS | cystathionine-beta-synthase |
| 78.4 | 1.190E-23 | 7.73E-20 | MAL | mal, T-cell differentiation protein |
| 40.8 | 1.420E-19 | 4.34E-17 | AQP8 | aquaporin 8 |
| 24.6 | 6.060E-16 | 6.6E-14 | NOXO1 | NADPH oxidase organizer 1 |
| 22.5 | 1.760E-14 | 1.24E-12 | CA4 | carbonic anhydrase IV |
| 18.0 | 3.580E-18 | 7.28E-16 | HOXA10 | homeobox A10 |
| 17.9 | 3.440E-20 | 1.4E-17 | SLC38A4 | solute carrier family 38, member 4 (SNAT4) |
| 15.8 | 2.110E-15 | 2.01E-13 | SLC26A4 | solute carrier family 26 (anion exchanger), member 4 |
| 15.1 | 5.970E-22 | 7.8E-19 | PON2 | paraoxonase 2 |
| 14.2 | 2.930E-21 | 2.55E-18 | SELENBP1 | selenium binding protein 1; selenium-binding protein 1-A-like |
| 14.2 | 4.790E-15 | 4.14E-13 | TFCP2L1 | transcription factor CP2-like 1 |
| 14.0 | 7.890E-15 | 6.28E-13 | ATP6V0D2 | ATPase, H+ transporting, lysosomal 38kDa, V0 subunit d2 |
| 13.2 | 3.340E-20 | 1.39E-17 | PADI3 | peptidyl arginine deiminase, type III |
| 12.9 | 5.970E-15 | 4.99E-13 | PLET1 | Placenta Expressed Transcript 1 |
| 12.9 | 3.030E-14 | 2.05E-12 | GJB2 | gap junction protein, beta 2, 26kDa |
| 12.0 | 8.160E-18 | 1.57E-15 | LY6E | lymphocyte antigen 6 complex, locus E-like |
| 11.2 | 8.740E-17 | 1.25E-14 | GSTA4 | glutathione S-transferase alpha 4 |
| 11.0 | 2.260E-23 | 1.03E-19 | B4GALNT3 | beta-1,4-N-acetyl-galactosaminyltransferase 3 |
| 10.4 | 1.160E-15 | 1.19E-13 | CERS4 | ceramide synthase 4 |
| 10.2 | 8.950E-19 | 2.23E-16 | CDHR1 | cadherin-related family member 1 |
| 9.8 | 9.030E-19 | 2.23E-16 | SPTSSB | serine palmitoyltransferase, small subunit B |
| 9.4 | 1.370E-12 | 5.37E-11 | CYP2W1 | cytochrome P450, family 2, subfamily W, polypeptide 1 |
| 9.3 | 1.440E-15 | 1.44E-13 | SCNN1A | sodium channel, non-voltage-gated 1 alpha subunit |
| 9.2 | 6.050E-16 | 6.6E-14 | ENDOD1 | endonuclease domain containing 1 |
| 9.0 | 1.210E-18 | 2.83E-16 | SH3BGRL2 | SH3 domain binding glutamic acid-rich protein like 2 |
| 8.9 | 3.790E-14 | 2.5E-12 | DSE | dermatan sulfate epimerase |
| 8.6 | 1.930E-17 | 3.51E-15 | SATB2 | SATB homeobox 2 |
| 8.6 | 2.840E-16 | 3.48E-14 | ATP6V0A4 | ATPase, H+ transporting, lysosomal V0 subunit a4 |
| 8.5 | 1.520E-16 | 2.01E-14 | INF2 | inverted formin, FH2 and WH2 domain containing |
| 8.3 | 8.030E-16 | 8.45E-14 | HOXA11 | Homeobox protein Hox-A11 |
| 8.2 | 1.430E-08 | 1.72E-07 | SLC26A3 | solute carrier family 26 (anion exchanger), member 3 |
| 8.1 | 2.330E-15 | 2.19E-13 | WDR72 | WD repeat domain 72 |
| 7.9 | 1.660E-10 | 3.52E-09 | GPRC5A | G protein-coupled receptor, family C, group 5, member A |
| 7.9 | 3.440E-15 | 3.09E-13 | ATP6V1G3 | ATPase, H+ transporting, lysosomal 13kDa, V1 subunit G3 |
| 7.6 | 1.020E-16 | 1.39E-14 | STMN2 | stathmin-like 2 |
| 7.4 | 3.550E-15 | 3.17E-13 | TLL2 | tolloid like 2 |
| 7.3 | 8.690E-14 | 4.88E-12 | SCNN1B | sodium channel, non voltage gated 1 beta subunit |
| 7.2 | 7.340E-14 | 4.3E-12 | NOV | nephroblastoma overexpressed |
| 6.8 | 4.820E-17 | 7.54E-15 | SFXN5 | sideroflexin 5 |
| 6.7 | 1.670E-13 | 8.62E-12 | SMOC1 | SPARC related modular calcium binding 1 |
| 6.6 | 2.580E-09 | 3.86E-08 | SPIC | Spi-C transcription factor (Spi-1/PU.1 related) |
| 6.5 | 1.390E-17 | 2.57E-15 | FAM101B | family with sequence similarity 101, member B |
| 6.4 | 5.760E-16 | 6.36E-14 | SLC16A14 | solute carrier family 16, member 14 |
| 6.3 | 1.860E-16 | 2.4E-14 | ABCC3 | ATP-binding cassette, sub-family C (CFTR/MRP), member 3 |
| 6.2 | 1.010E-11 | 3.07E-10 | TSPAN7 | tetraspanin 7 |
| 6.2 | 2.830E-20 | 1.23E-17 | HIGD1A | HIG1 domain family member 1A, mitochondrial |
| 6.1 | 1.060E-09 | 1.75E-08 | FNDC1 | fibronectin type III domain containing 1 |
| 6.1 | 4.620E-17 | 7.3E-15 | PAPSS2 | 3-phosphoadenosine 5-phosphosulfate synthase 2 |
| 5.8 | 1.890E-20 | 8.64E-18 | HOXD8 | Homeobox protein Hox-D8 |
| 5.7 | 3.830E-13 | 1.77E-11 | BEST4 | bestrophin 4 |
| 5.7 | 4.320E-14 | 2.78E-12 | VILL | villin-like |
| 5.6 | 1.650E-10 | 3.51E-09 | LUM | lumican |
| 5.5 | 6.580E-20 | 2.19E-17 | HOXD3 | homeobox D3 |
| 5.4 | 8.650E-13 | 3.61E-11 | KCNJ15 | potassium inwardly-rectifying channel, subfamily J, member 15 |
| 5.4 | 4.700E-13 | 2.12E-11 | EMP1 | epithelial membrane protein 1 |
| 5.4 | 5.460E-20 | 1.96E-17 | LRRC42 | leucine rich repeat containing 42 |
| 5.3 | 2.870E-15 | 2.64E-13 | CHRDL1 | chordin-like 1 |
| 5.3 | 3.620E-16 | 4.31E-14 | GLIPR2 | GLI pathogenesis-related 2 |
| 5.3 | 1.140E-09 | 1.87E-08 | OGN | osteoglycin |
| 5.2 | 3.450E-14 | 2.31E-12 | HOXA10-AS | HOXA10 antisense RNA |
| 5.1 | 3.650E-13 | 1.71E-11 | SCNN1G | sodium channel, non voltage gated 1 gamma subunit |
| 5.1 | 7.630E-13 | 3.22E-11 | SCD | stearoyl-CoA desaturase (delta-9-desaturase) |
| 5.0 | 3.730E-16 | 4.37E-14 | LDHB | lactate dehydrogenase B |
| 5.0 | 4.410E-17 | 7.09E-15 | HOXD9 | homeobox D9; homeobox protein Hox-D10 |
| 4.9 | 9.120E-17 | 1.28E-14 | STEAP3 | STEAP family member 3, metalloreductase |
| 4.9 | 2.260E-18 | 5.04E-16 | SEMA3G | sema domain, immunoglobulin domain (Ig), short basic domain, secreted, (semaphorin) 3G |
| 4.9 | 3.520E-17 | 5.81E-15 | FRAS1 | Fraser extracellular matrix complex subunit 1 |
| 4.8 | 1.070E-14 | 8.17E-13 | EPHB3 | EPH receptor B3 |
| 4.8 | 3.260E-13 | 1.55E-11 | GUCY1A2 | guanylate cyclase 1, soluble, alpha 2 |
| 4.7 | 2.150E-15 | 2.04E-13 | FRY | furry homolog (Drosophila) |
| 4.7 | 1.020E-16 | 1.39E-14 | LIPG | lipase, endothelial |
| 4.7 | 3.140E-16 | 3.75E-14 | HOXD4 | homeobox D4 |
| 4.7 | 2.750E-17 | 4.76E-15 | APITD1 | apoptosis-inducing, TAF9-like domain 1 |
| 4.7 | 2.720E-08 | 3.02E-07 | ADH1C | alcohol dehydrogenase 1C (class I), gamma polypeptide |
| 4.7 | 4.750E-15 | 4.12E-13 | MPST | mercaptopyruvate sulfurtransferase |
| 4.6 | 3.980E-15 | 3.51E-13 | LRP8 | low density lipoprotein receptor-related protein 8 |
| 4.6 | 8.370E-12 | 2.58E-10 | ITGB6 | integrin, beta 6 |
| 4.6 | 3.220E-10 | 6.25E-09 | ACTA2 | actin, alpha 2, smooth muscle, aorta |
| 4.5 | 4.920E-15 | 4.21E-13 | HK1 | hexokinase 1 |
| 4.5 | 5.020E-09 | 6.87E-08 | ABI3BP | ABI family, member 3 (NESH) binding protein |
| 4.5 | 1.410E-12 | 5.46E-11 | VNN1 | vanin 1 |
| 4.5 | 1.650E-14 | 1.18E-12 | HK2 | hexokinase-2 |
| 4.4 | 2.690E-18 | 5.8E-16 | HSPB8 | heat shock 22kDa protein 8 |
| 4.3 | 1.110E-10 | 2.47E-09 | FAM26E | family with sequence similarity 26, member E |
| 4.3 | 1.340E-14 | 9.71E-13 | POSTN | periostin, osteoblast specific factor |
| 4.3 | 8.680E-06 | 4.72E-05 | LYGL | lysozyme g-like |
| 4.3 | 8.640E-14 | 4.87E-12 | DIO2 | deiodinase, iodothyronine, type II |
| 4.2 | 1.270E-10 | 2.79E-09 | NRG1 | neuregulin 1 |
| 4.2 | 5.020E-14 | 3.19E-12 | HOXA9 | homeobox A9; homeobox protein Hox-A9-like |
| 4.2 | 1.500E-10 | 3.24E-09 | KPNA2 | karyopherin alpha 2 (RAG cohort 1, importin alpha 1) |
| 4.2 | 2.620E-18 | 5.7E-16 | PPARD | peroxisome proliferator-activated receptor delta |
| 4.1 | 1.480E-12 | 5.69E-11 | WLS | wntless Wnt ligand secretion mediator |
| 4.1 | 1.010E-09 | 1.69E-08 | S100B | S100 calcium binding protein B |
| 4.1 | 1.600E-14 | 1.15E-12 | DDAH1 | dimethylarginine dimethylaminohydrolase 1 |
| 4.1 | 1.710E-13 | 8.76E-12 | PRDX6 | peroxiredoxin 6 |
| 4.0 | 1.390E-13 | 7.36E-12 | CDCA7L | cell division cycle associated 7-like |
| 4.0 | 3.760E-13 | 1.75E-11 | SALL1 | sal-like 1 (Drosophila) |
| 4.0 | 1.600E-15 | 1.59E-13 | ATP6V1C2 | ATPase, H+ transporting, lysosomal 42kDa, V1 subunit C2 |
| 4.0 | 2.080E-12 | 7.58E-11 | CMBL | carboxymethylenebutenolidase homolog (Pseudomonas) |
| 4.0 | 2.320E-16 | 2.89E-14 | BAG3 | BCL2-associated athanogene 3 |
| 4.0 | 2.200E-17 | 3.87E-15 | TXN | thioredoxin |
| 4.0 | 5.620E-13 | 2.47E-11 | BMP7 | bone morphogenetic protein 7 |
| 4.0 | 2.320E-11 | 6.38E-10 | GLDC | glycine dehydrogenase (decarboxylating) |
| 3.9 | 2.970E-15 | 2.71E-13 | TFRC | transferrin receptor (p90, CD71) |
| 3.9 | 8.590E-12 | 2.63E-10 | MYB | v-myb avian myeloblastosis viral oncogene homolog |
| 3.9 | 8.950E-13 | 3.71E-11 | FAM83D | family with sequence similarity 83, member D |
| 3.9 | 5.300E-11 | 1.3E-09 | KCNG3 | potassium channel, voltage gated modifier subfamily G, member 3 |
| 3.9 | 1.930E-13 | 9.79E-12 | TRPA1 | transient receptor potential cation channel, subfamily A, member 1 |
| 3.8 | 1.420E-08 | 1.72E-07 | AQP1 | ACQUAPORIN 1 |
| 3.8 | 5.120E-07 | 3.87E-06 | C4BPA | complement component 4 binding protein, alpha |
| 3.8 | 4.930E-08 | 5.05E-07 | PLK1 | polo-like kinase 1 |
| 3.8 | 7.380E-08 | 7.21E-07 | MKI67 | marker of proliferation Ki-67 |
| 3.8 | 9.830E-15 | 7.69E-13 | SESTD1 | SEC14 and spectrin domains 1 |
| 3.8 | 1.950E-12 | 7.24E-11 | PPARG | peroxisome proliferator-activated receptor gamma |
| 3.8 | 6.460E-14 | 3.88E-12 | HOXA6 | homeobox A6; homeobox protein Hox-A6-like |
| 3.8 | 5.800E-12 | 1.89E-10 | MOXD1 | monooxygenase, DBH |
| 3.8 | 9.440E-14 | 5.26E-12 | NDE1 | nudE neurodevelopment protein 1 |
| 3.7 | 2.520E-11 | 6.82E-10 | GEM | GTP binding protein overexpressed in skeletal muscle |
| 3.7 | 2.970E-13 | 1.43E-11 | PAQR5 | progestin and adipoQ receptor family member V |
| 3.7 | 8.520E-13 | 3.56E-11 | LINGO1 | leucine rich repeat and Ig domain containing 1 |
| 3.6 | 2.220E-12 | 8.07E-11 | EPHB2 | EPH receptor B2 |
| 3.6 | 1.280E-17 | 2.41E-15 | KCNQ1 | potassium channel, voltage gated KQT-like subfamily Q, member 1 |
| 3.6 | 4.370E-17 | 7.08E-15 | FAM3D | family with sequence similarity 3, member D |
| 3.6 | 1.020E-14 | 7.85E-13 | GPR85 | G protein-coupled receptor 85 |
| 3.6 | 9.100E-12 | 2.78E-10 | HS3ST3B1L | heparan sulfate glucosamine 3-O-sulfotransferase 3B1-like |
| 3.6 | 6.020E-09 | 8.09E-08 | CCNB2 | cyclin B2 |
| 3.6 | 3.050E-16 | 3.67E-14 | FABP3 | fatty acid binding protein 3, muscle and heart (mammary-derived growth inhibitor) |
| 3.6 | 4.700E-13 | 2.12E-11 | LYPD6B | LY6/PLAUR domain containing 6B |
| 3.6 | 1.750E-09 | 2.74E-08 | CA7 | carbonic anhydrase VII |
| 3.6 | 1.830E-11 | 5.15E-10 | SVEP1 | sushi, von Willebrand factor type A, EGF and pentraxin domain containing 1 |
| 3.6 | 5.100E-11 | 1.26E-09 | GNG4 | guanine nucleotide binding protein (G protein), gamma 4 |
| 3.6 | 6.290E-14 | 3.8E-12 | NAALADL2 | N-acetylated alpha-linked acidic dipeptidase-like 2 |
| 3.5 | 1.980E-12 | 7.31E-11 | FOXI1 | forkhead box I1 |
| 3.5 | 7.870E-09 | 1.02E-07 | KIF4A | kinesin family member 4A |
| 3.5 | 5.630E-12 | 1.85E-10 | IGFBP5 | Insulin Like Growth Factor Binding Protein 5 |
| 3.5 | 3.000E-10 | 5.86E-09 | CYGB | cytoglobin |
| 3.5 | 2.800E-10 | 5.52E-09 | hoxa13 | homeobox A13 |
| 3.5 | 5.680E-14 | 3.51E-12 | ZBTB10 | zinc finger and BTB domain containing 10 |
| 3.5 | 1.000E-04 | 0.0005 | GAL3ST2 | galactose-3-O-sulfotransferase 2 |
| 3.5 | 4.530E-13 | 2.07E-11 | ADAMTS1 | ADAM metallopeptidase with thrombospondin type 1 motif, 1 |
| 3.5 | 1.260E-10 | 2.78E-09 | AKR1B10 | aldo-keto reductase family 1, member B10 (aldose reductase) |
| 3.5 | 8.680E-07 | 6.13E-06 | GSTT1L | glutathione S-transferase theta 1-like |
| 3.5 | 2.640E-13 | 1.29E-11 | SASH1 | SAM and SH3 domain containing 1 |
| 3.4 | 4.930E-12 | 1.65E-10 | SLITRK2 | SLIT and NTRK-like family, member 2 |
| 3.4 | 2.820E-13 | 1.36E-11 | ATP8A2 | ATPase, aminophospholipid transporter, class I, type 8A, member 2 |
| 3.4 | 1.340E-11 | 3.91E-10 | AURKA | aurora kinase A |
| 3.4 | 1.100E-11 | 3.28E-10 | FRZB | frizzled-related protein |
| 3.4 | 4.720E-11 | 1.18E-09 | DUSP14 | dual specificity phosphatase 14 |
| 3.4 | 5.280E-09 | 7.18E-08 | THY1 | Thy-1 cell surface antigen |
| 3.4 | 3.180E-09 | 4.64E-08 | LAMB1 | laminin, beta 1 |
| 3.4 | 4.570E-12 | 1.55E-10 | RXFP1 | relaxin/insulin-like family peptide receptor 1 |
| 3.4 | 6.980E-11 | 1.65E-09 | BMP4 | bone morphogenetic protein 4 |
| 3.4 | 1.220E-09 | 1.99E-08 | ROBO1 | roundabout, axon guidance receptor, homolog 1 (Drosophila) |
| 3.4 | 3.350E-11 | 8.71E-10 | CSRP1 | cysteine and glycine-rich protein 1 |
| 3.4 | 9.530E-13 | 3.89E-11 | SLC2A1 | solute carrier family 2 (facilitated glucose transporter), member 1 |
| 3.4 | 4.720E-13 | 2.12E-11 | ETV4 | ETS translocation variant 4-like; ets variant 4 |
| 3.4 | 1.880E-11 | 5.27E-10 | NCAM1 | neural cell adhesion molecule 1 |
| 3.3 | 2.910E-12 | 1.03E-10 | GPRC6A | G protein-coupled receptor, class C, group 6, member A |
| 3.3 | 2.660E-13 | 1.29E-11 | PGD | phosphogluconate dehydrogenase |
| 3.3 | 3.680E-08 | 3.9E-07 | BUB1 | BUB1 mitotic checkpoint serine/threonine kinase |
| 3.3 | 2.900E-08 | 3.18E-07 | TAGLN | Transgelin |
| 3.3 | 7.250E-14 | 4.27E-12 | SLC8A1 | solute carrier family 8 (sodium/calcium exchanger), member 1 |
| 3.3 | 4.960E-15 | 4.23E-13 | HDAC1 | histone deacetylase 1 |
| 3.3 | 2.170E-13 | 1.08E-11 | RNF223 | ring finger protein 223 |
| 3.3 | 3.430E-10 | 6.64E-09 | TTC38 | tetratricopeptide repeat domain 38 |
| 3.3 | 8.100E-14 | 4.62E-12 | ESYT3 | extended synaptotagmin-like protein 3 |
| 3.3 | 6.800E-09 | 9.01E-08 | ifitm1 | interferon-induced transmembrane protein 3-like |
| 3.3 | 4.610E-13 | 2.1E-11 | WIF1 | WNT inhibitory factor 1 |
| 3.2 | 1.240E-09 | 2.01E-08 | VIPR2 | vasoactive intestinal peptide receptor 2 |
| 3.2 | 5.230E-11 | 1.28E-09 | CTGF | connective tissue growth factor |
| 3.2 | 2.050E-16 | 2.61E-14 | ARHGEF10L | Rho guanine nucleotide exchange factor (GEF) 10-like |
| 3.2 | 3.870E-14 | 2.54E-12 | ATP2A3 | ATPase, Ca++ transporting, ubiquitous |
| 3.2 | 2.100E-17 | 3.73E-15 | CA9 | carbonic anhydrase IX |
| 3.2 | 4.050E-10 | 7.64E-09 | PHLDA2 | pleckstrin homology-like domain, family A, member 2 |
| 3.2 | 1.030E-08 | 1.29E-07 | TOP2A | topoisomerase (DNA) II alpha 170kDa |
| 3.2 | 3.390E-07 | 2.7E-06 | USP18 | ubiquitin specific peptidase 18 |
| 3.2 | 1.110E-15 | 1.15E-13 | SMS | spermine synthase |
| 3.2 | 3.800E-11 | 9.69E-10 | ANXA1 | annexin A1 |
| 3.2 | 6.210E-12 | 2E-10 | ASL2 | argininosuccinate lyase |
| 3.2 | 2.590E-09 | 3.87E-08 | CYR61 | cysteine-rich, angiogenic inducer, 61 |
| 3.2 | 7.090E-10 | 1.25E-08 | WNT5A | wingless-type MMTV integration site family, member 5A |
| 3.2 | 4.120E-07 | 3.2E-06 | CRISPLD2 | cysteine-rich secretory protein LCCL domain containing 2 |
| 3.2 | 6.050E-15 | 5.03E-13 | HSPB11 | heat shock protein family B (small), member 11 |
| 3.2 | 4.910E-10 | 8.98E-09 | LGR5 | leucine-rich repeat containing G protein-coupled receptor 5 |
| 3.2 | 6.220E-13 | 2.69E-11 | UGDH | UDP-glucose 6-dehydrogenase |
| 3.1 | 6.130E-09 | 8.22E-08 | RRM2 | ribonucleotide reductase M2 |
| 3.1 | 1.340E-10 | 2.93E-09 | ECT2 | epithelial cell transforming 2 |
| 3.1 | 5.520E-10 | 9.94E-09 | ITGA8 | integrin, alpha 8 |
| 3.1 | 1.740E-11 | 4.95E-10 | LGALS1 | lectin, galactoside-binding, soluble, 1 |
| 3.1 | 1.030E-13 | 5.7E-12 | DIAPH3 | diaphanous-related formin 3 |
| 3.1 | 4.010E-07 | 3.12E-06 | TNFAIP2 | tumor necrosis factor, alpha-induced protein 2 |
| 3.1 | 1.360E-11 | 3.96E-10 | MLLT3 | myeloid/lymphoid or mixed-lineage leukemia translocated to chromosome 3 |
| 3.1 | 5.300E-09 | 7.2E-08 | TUBA3E | tubulin, alpha 3e; anche introne di csrp1 (Cysteine and glycine-rich protein 1) |
| 3.1 | 3.190E-11 | 8.34E-10 | MET | met proto-oncogene; hepatocyte growth factor receptor-like |
| 3.1 | 5.870E-08 | 5.87E-07 | GTSE1 | G2 and S phase-expressed protein 1 |
| 3.1 | 3.740E-12 | 1.3E-10 | CA2 | carbonic anhydrase II |
| 3.1 | 6.690E-12 | 2.14E-10 | NUF2 | NUF2, NDC80 kinetochore complex component |
| 3.1 | 1.500E-11 | 4.31E-10 | OSR1 | odd-skipped related transciption factor 1 |
| 3.1 | 2.570E-11 | 6.96E-10 | PARVB | parvin, beta |
| 3.0 | 3.250E-13 | 1.55E-11 | B3GAT2 | beta-1,3-glucuronyltransferase 2 |
| 3.0 | 4.600E-14 | 2.94E-12 | SP8 | Sp8 transcription factor |
| 3.0 | 4.710E-12 | 1.59E-10 | CCDC80 | coiled-coil domain containing 80 |
| 3.0 | 2.330E-05 | 0.0001 | CXCR5 | chemokine (C-X-C motif) receptor 5 |
| 3.0 | 2.710E-14 | 1.85E-12 | FANCA | Fanconi anemia, complementation group A |
| 3.0 | 2.040E-12 | 7.48E-11 | GLIPR1L | GLI pathogenesis-related 1-like |
| 3.0 | 3.510E-07 | 2.79E-06 | IL8L1 | interleukin 8-like 1 |
| 3.0 | 1.050E-09 | 1.74E-08 | CAPN5 | calpain 5 |
| 3.0 | 3.550E-08 | 3.79E-07 | HOXA3 | homeobox A3 |
| 3.0 | 1.540E-11 | 4.43E-10 | FGF7 | fibroblast growth factor 7 |
| 3.0 | 6.640E-10 | 1.18E-08 | RRM1 | ribonucleotide reductase M1 |
| 3.0 | 2.400E-10 | 4.83E-09 | RBPMS2 | RNA binding protein with multiple splicing 2 |
| 3.0 | 3.290E-05 | 0.0002 | RSAD2 | radical S-adenosyl methionine domain containing 2 |
| 3.0 | 6.350E-15 | 5.23E-13 | TXNRD3 | thioredoxin reductase 3 |
| 3.0 | 3.640E-15 | 3.22E-13 | ANKRD22 | ankyrin repeat domain 22 |
| 3.0 | 2.200E-11 | 6.07E-10 | TTK | TTK protein kinase |
| 3.0 | 1.960E-14 | 1.37E-12 | UNC119 | unc-119 homolog (C. elegans) |
| 3.0 | 5.610E-17 | 8.67E-15 | PLA2G4F | phospholipase A2, group IVF; vacuolar protein sorting 39 homolog (S. cerevisiae) |
| 3.0 | 4.020E-10 | 7.57E-09 | SMC2 | structural maintenance of chromosomes 2 |
| 3.0 | 2.400E-06 | 1.51E-05 | AVD | Avidin |
| 3.0 | 6.840E-08 | 6.76E-07 | CASC5 | cancer susceptibility candidate 5 |
| 3.0 | 2.090E-08 | 2.4E-07 | TPX2 | TPX2, microtubule-associated |
| 2.9 | 2.770E-11 | 7.38E-10 | RXFP2 | relaxin/insulin-like family peptide receptor 2,) |
| 2.9 | 2.660E-13 | 1.29E-11 | SULT1E1 | sulfotransferase family 1E, estrogen-preferring, member 1 |
| 2.9 | 3.670E-09 | 5.27E-08 | VWF | von Willebrand factor |
| 2.9 | 4.090E-09 | 5.76E-08 | ADAMTS15 | ADAM metallopeptidase with thrombospondin type 1 motif, 15 |
| 2.9 | 1.690E-07 | 1.49E-06 | B3GALT5 | UDP-Gal:betaGlcNAc beta 1,3-galactosyltransferase, polypeptide 5 |
| 2.9 | 1.010E-06 | 7.01E-06 | PDGFD | platelet derived growth factor D |
| 2.9 | 1.780E-11 | 5.04E-10 | PSD3 | pleckstrin and Sec7 domain containing 3 |
| 2.9 | 3.190E-12 | 1.12E-10 | ETV1 | ets variant 1 |
| 2.9 | 4.890E-13 | 2.17E-11 | MID1 | midline 1 (Opitz/BBB syndrome) |
| 2.9 | 2.870E-07 | 2.35E-06 | NUSAP1 | nucleolar and spindle associated protein 1 |
| 2.9 | 6.820E-17 | 1.02E-14 | AOX1 | aldehyde oxidase 1 |
| 2.9 | 5.130E-10 | 9.31E-09 | ARL14 | ADP-ribosylation factor-like protein 14 |
| 2.9 | 6.850E-14 | 4.07E-12 | FAM155B | family with sequence similarity 155, member B |
| 2.9 | 1.450E-13 | 7.63E-12 | FAR1 | fatty acyl CoA reductase 1 |
| 2.9 | 1.800E-11 | 5.1E-10 | ADAMTSL2 | ADAMTS-like 2 |
| 2.9 | 3.820E-11 | 9.7E-10 | APOH | apolipoprotein H (beta-2-glycoprotein I) |
| 2.9 | 5.150E-13 | 2.27E-11 | EPB41L2 | erythrocyte membrane protein band 4.1-like 2 |
| 2.9 | 6.320E-09 | 8.42E-08 | HMMR | hyaluronan-mediated motility receptor (RHAMM) |
| 2.9 | 3.320E-14 | 2.23E-12 | HOMER1 | homer scaffolding protein 1 |
| 2.9 | 2.780E-18 | 5.92E-16 | HOXB9 | homeobox B9 |
| 2.9 | 1.510E-16 | 2.01E-14 | ERO1L | ERO1-like (S. cerevisiae) |
| 2.9 | 3.670E-08 | 3.9E-07 | AIFM2 | apoptosis-inducing factor, mitochondrion-associated, 2 |
| 2.9 | 6.090E-13 | 2.64E-11 | ABCC1 | ATP-binding cassette, sub-family C (CFTR/MRP), member 1 |
| 2.9 | 6.440E-06 | 3.61E-05 | CYYR1 | cysteine/tyrosine-rich 1 |
| 2.9 | 1.520E-12 | 5.8E-11 | KHDRBS3 | KH domain containing, RNA binding, signal transduction associated 3 |
| 2.9 | 3.700E-14 | 2.45E-12 | PLEKHA6 | pleckstrin homology domain containing, family A member 6 |
| 2.8 | 3.770E-13 | 1.75E-11 | FAM20A | family with sequence similarity 20, member A |
| 2.8 | 3.640E-14 | 2.42E-12 | SLC16A3 | solute carrier family 16 (monocarboxylate transporter), member 3 |
| 2.8 | 2.930E-09 | 4.3E-08 | CKAP2 | cytoskeleton associated protein 2 |
| 2.8 | 5.130E-12 | 1.71E-10 | CTNND2 | catenin (cadherin-associated protein), delta 2 |
| 2.8 | 1.350E-13 | 7.19E-12 | EML1 | echinoderm microtubule associated protein like 1 |
| 2.8 | 1.140E-15 | 1.18E-13 | ICA1 | islet cell autoantigen 1, 69kDa |
| 2.8 | 2.500E-12 | 8.96E-11 | KIAA1210 | KIAA1210 |
| 2.8 | 1.080E-13 | 5.93E-12 | BEND6 | BEN domain containing 6 |
| 2.8 | 1.560E-09 | 2.48E-08 | CTH | cystathionine gamma-lyase |
| 2.8 | 2.800E-08 | 3.09E-07 | SYNPO2 | synaptopodin 2 |
| 2.8 | 7.520E-15 | 6.06E-13 | SLC16A1 | solute carrier family 16 (monocarboxylate transporter), member 1 |
| 2.8 | 4.290E-09 | 5.98E-08 | LMNB2 | lamin B2 |
| 2.8 | 1.050E-08 | 1.31E-07 | ATP10B | ATPase, class V, type 10B |
| 2.8 | 2.450E-12 | 8.81E-11 | CCDC60 | coiled-coil domain containing 60 |
| 2.8 | 7.390E-11 | 1.73E-09 | CDK1 | cyclin-dependent kinase 1 |
| 2.8 | 3.260E-13 | 1.55E-11 | EMILIN2 | elastin microfibril interfacer 2 |
| 2.8 | 7.570E-12 | 2.38E-10 | FSTL4 | follistatin-like 4 |
| 2.8 | 8.320E-11 | 1.92E-09 | MPP6 | membrane protein, palmitoylated 6 (MAGUK p55 subfamily member 6) |
| 2.8 | 3.820E-06 | 2.27E-05 | MXRA5 | matrix-remodelling associated 5 |
| 2.8 | 1.620E-10 | 3.46E-09 | ANLN | anillin actin binding protein |
| 2.8 | 2.070E-08 | 2.37E-07 | ASS1 | argininosuccinate synthase |
| 2.8 | 8.030E-17 | 1.16E-14 | MYO10L | myosin-X-like |
| 2.7 | 4.610E-08 | 4.77E-07 | CENPF | centromere protein F, 350/400kDa |
| 2.7 | 2.080E-07 | 1.77E-06 | FGFBP1 | fibroblast growth factor binding protein 1 |
| 2.7 | 1.150E-08 | 1.42E-07 | CDH13 | cadherin 13 |
| 2.7 | 5.460E-12 | 1.81E-10 | LPCAT2 | lysophosphatidylcholine acyltransferase 2 |
| 2.7 | 5.940E-09 | 8.01E-08 | FHL2 | four and a half LIM domains 2 |
| 2.7 | 1.510E-10 | 3.26E-09 | SEMA3A | sema domain, immunoglobulin domain (Ig), short basic domain, secreted, (semaphorin) 3A |
| 2.7 | 1.390E-06 | 9.3E-06 | CXCL13L2 | chemokine |
| 2.7 | 1.630E-15 | 1.61E-13 | GMDS | GDP-mannose 4,6-dehydratase |
| 2.7 | 2.510E-08 | 2.8E-07 | KIF20A | kinesin family member 20A |
| 2.7 | 9.360E-09 | 1.19E-07 | NDC80 | NDC80 kinetochore complex component |
| 2.7 | 6.510E-11 | 1.56E-09 | PBK | PDZ binding kinase |
| 2.7 | 4.410E-09 | 6.12E-08 | TPBG | trophoblast glycoprotein |
| 2.7 | 1.670E-09 | 2.63E-08 | CDC20 | cell division cycle 20 |
| 2.7 | 8.360E-12 | 2.58E-10 | HSBP1L1 | heat shock factor binding protein 1-like 1 |
| 2.7 | 2.270E-10 | 4.61E-09 | TMEM213 | transmembrane protein 213 |
| 2.7 | 2.720E-11 | 7.28E-10 | CHPT1 | choline phosphotransferase 1 |
| 2.7 | 1.190E-11 | 3.51E-10 | PLAC9 | placenta-specific 9 |
| 2.7 | 2.220E-11 | 6.13E-10 | PYCR1 | pyrroline-5-carboxylate reductase 2 |
| 2.7 | 4.980E-08 | 5.1E-07 | ARHGEF39 | Rho guanine nucleotide exchange factor (GEF) 39 |
| 2.7 | 1.890E-09 | 2.92E-08 | BUB1B | BUB1 mitotic checkpoint serine/threonine kinase B |
| 2.7 | 2.900E-13 | 1.4E-11 | METRNL | meteorin, glial cell differentiation regulator-like |
| 2.7 | 8.220E-09 | 1.06E-07 | PLK4 | polo-like kinase 4 |
| 2.7 | 3.160E-12 | 1.11E-10 | ST5 | suppression of tumorigenicity 5 |
| 2.7 | 3.280E-08 | 3.54E-07 | MYBL1 | v-myb avian myeloblastosis viral oncogene homolog-like 1 |
| 2.7 | 4.680E-09 | 6.47E-08 | RACGAP1 | Rac GTPase activating protein 1 |
| 2.7 | 4.680E-10 | 8.62E-09 | TPH1 | tryptophan hydroxylase 1 |
| 2.6 | 4.700E-13 | 2.12E-11 | ATOH8 | protein atonal homolog 8 |
| 2.6 | 1.400E-12 | 5.43E-11 | TPST1 | tyrosylprotein sulfotransferase 1 |
| 2.6 | 3.520E-10 | 6.76E-09 | arsh | arylsulfatase family, member H |
| 2.6 | 4.770E-13 | 2.14E-11 | MIF | macrophage migration inhibitory factor (glycosylation-inhibiting factor) |
| 2.6 | 5.110E-08 | 5.2E-07 | GCNT4 | glucosaminyl (N-acetyl) transferase 4, core 2 |
| 2.6 | 5.410E-11 | 1.32E-09 | TSPO | translocator protein (18kDa) |
| 2.6 | 1.680E-10 | 3.55E-09 | BORA | bora, aurora kinase A activator |
| 2.6 | 1.060E-11 | 3.19E-10 | CLCNKB | chloride channel, voltage-sensitive Kb |
| 2.6 | 1.240E-11 | 3.67E-10 | INTU | inturned planar cell polarity protein |
| 2.6 | 2.160E-12 | 7.89E-11 | PPP1R9A | protein phosphatase 1, regulatory subunit 9A |
| 2.6 | 1.270E-13 | 6.82E-12 | AMD1 | adenosylmethionine decarboxylase 1 |
| 2.6 | 2.210E-09 | 3.37E-08 | SLC16A5 | solute carrier family 16 (monocarboxylate transporter), member 5 |
| 2.6 | 1.620E-09 | 2.56E-08 | KIF2C | kinesin-like protein KIF2C-like; kinesin family member 2C |
| 2.6 | 1.630E-06 | 1.07E-05 | ME1 | malic enzyme 1, NADP(+)-dependent, cytosolic |
| 2.6 | 1.360E-10 | 2.96E-09 | SERTAD4 | SERTA domain containing 4 |
| 2.6 | 7.070E-06 | 3.93E-05 | COL6A2 | collagen, type VI, alpha 2 |
| 2.6 | 1.820E-10 | 3.8E-09 | KLF5 | Kruppel-like factor 5 (intestinal) |
| 2.6 | 3.100E-07 | 2.5E-06 | NEK2 | NIMA-related kinase 2 |
| 2.5 | 1.310E-12 | 5.16E-11 | AGPAT5 | 1-acylglycerol-3-phosphate O-acyltransferase 5 |
| 2.5 | 3.510E-08 | 3.76E-07 | KIF15 | kinesin family member 15 |
| 2.5 | 1.820E-10 | 3.8E-09 | LPHN2 | latrophilin 2 |
| 2.5 | 1.660E-11 | 4.72E-10 | CDC7 | cell division cycle 7 |
| 2.5 | 1.440E-09 | 2.31E-08 | NEXN | nexilin (F actin binding protein) |
| 2.5 | 1.300E-10 | 2.85E-09 | SMOC2 | SPARC related modular calcium binding 2 |
| 2.5 | 1.050E-09 | 1.74E-08 | AHNAK2 | protein AHNAK2-like |
| 2.5 | 5.930E-06 | 3.35E-05 | MGP | matrix Gla protein |
| 2.5 | 4.290E-14 | 2.78E-12 | PTDSS2 | phosphatidylserine synthase 2 |
| 2.5 | 8.470E-11 | 1.95E-09 | CEP55 | centrosomal protein 55kDa |
| 2.5 | 1.160E-07 | 1.07E-06 | ASPM | asp (abnormal spindle) homolog, microcephaly associated (Drosophila) |
| 2.5 | 5.660E-11 | 1.37E-09 | GREM2 | gremlin 2, DAN family BMP antagonist |
| 2.5 | 2.000E-12 | 7.35E-11 | HOXA4 | homeobox A4; homeobox protein Hox-A4-like |
| 2.5 | 1.270E-10 | 2.79E-09 | MLF1 | myeloid leukemia factor 1 |
| 2.5 | 2.710E-06 | 1.68E-05 | ACTG2 | Actin, gamma-enteric smooth muscle |
| 2.5 | 1.280E-05 | 0.000066 | AKAP12 | A kinase (PRKA) anchor protein 12 |
| 2.5 | 4.220E-09 | 5.89E-08 | CBLN1 | cerebellin 1 precursor |
| 2.5 | 2.420E-10 | 4.87E-09 | ELOVL6 | ELOVL fatty acid elongase 6 |
| 2.5 | 2.050E-07 | 1.76E-06 | MCM5 | minichromosome maintenance complex component 5 |
| 2.5 | 1.830E-05 | 9.12E-05 | PAX5 | paired box 5 |
| 2.5 | 5.800E-13 | 2.54E-11 | RAPGEF5 | Rap guanine nucleotide exchange factor (GEF) 5 |
| 2.5 | 2.460E-12 | 8.84E-11 | CLSTN1 | calsyntenin 1 |
| 2.5 | 4.930E-08 | 5.05E-07 | DLGAP5 | discs, large (Drosophila) homolog-associated protein 5 |
| 2.5 | 1.880E-10 | 3.91E-09 | ADAMTS5 | ADAM metallopeptidase with thrombospondin type 1 motif, 5 |
| 2.5 | 3.060E-10 | 5.98E-09 | CTNS | cystinosin, lysosomal cystine transporter |
| 2.5 | 3.020E-11 | 7.97E-10 | SPC25 | SPC25, NDC80 kinetochore complex component |
| 2.5 | 3.330E-09 | 4.83E-08 | EAF2 | ELL associated factor 2 |
| 2.5 | 1.060E-13 | 5.84E-12 | GOLM1 | golgi membrane protein 1 |
| 2.5 | 7.650E-08 | 7.44E-07 | MCM3 | minichromosome maintenance complex component 3 |
| 2.5 | 7.310E-13 | 3.09E-11 | C16ORF45 | chromosome 14 open reading frame, human C16orf45 |
| 2.5 | 9.080E-09 | 1.16E-07 | CENPK | centromere protein K |
| 2.5 | 8.390E-07 | 5.95E-06 | CXCL12 | chemokine (C-X-C motif) ligand 12 |
| 2.5 | 4.160E-09 | 5.83E-08 | DACT2 | dishevelled-binding antagonist of beta-catenin 2 |
| 2.5 | 1.250E-08 | 1.54E-07 | FAM3B | family with sequence similarity 3, member B |
| 2.5 | 3.740E-09 | 5.35E-08 | LAMA5 | laminin, alpha 5 |
| 2.5 | 4.930E-09 | 6.78E-08 | MELK | maternal embryonic leucine zipper kinase; maternal embryonic leucine zipper kinase-like |
| 2.5 | 6.740E-06 | 3.76E-05 | MYH11 | Myosin-11 |
| 2.5 | 2.650E-11 | 7.13E-10 | NMRAL1 | NmrA-like family domain containing 1 |
| 2.5 | 8.490E-09 | 1.09E-07 | TMEM248 | transmembrane protein 248 |
| 2.5 | 4.900E-07 | 3.72E-06 | VCAN | versican core protein precursor |
| 2.4 | 7.220E-15 | 5.87E-13 | ENAH | enabled homolog (Drosophila) |
| 2.4 | 2.710E-06 | 1.67E-05 | HVCN1 | hydrogen voltage gated channel 1 |
| 2.4 | 1.790E-09 | 2.79E-08 | PRC1 | protein regulator of cytokinesis 1 |
| 2.4 | 1.330E-11 | 3.88E-10 | TACC3 | transforming, acidic coiled-coil containing protein 3 |
| 2.4 | 9.170E-11 | 2.09E-09 | CENPL | centromere protein L |
| 2.4 | 1.370E-08 | 1.66E-07 | S100A11 | S100 calcium binding protein A11 |
| 2.4 | 8.420E-10 | 1.45E-08 | CLMN | calmin (calponin-like, transmembrane) |
| 2.4 | 2.770E-14 | 1.88E-12 | PARM1 | prostate androgen-regulated mucin-like protein 1 |
| 2.4 | 7.670E-14 | 4.42E-12 | WISP1 | WNT1 inducible signaling pathway protein 1 |
| 2.4 | 1.540E-13 | 7.99E-12 | ENY2 | enhancer of yellow 2 homolog (Drosophila) |
| 2.4 | 3.520E-12 | 1.23E-10 | FASN | fatty acid synthase; serine/arginine repetitive matrix protein 2-like |
| 2.4 | 3.780E-11 | 9.66E-10 | PAICS | phosphoribosylaminoimidazole carboxylase, phosphoribosylaminoimidazole succinocarboxamide synthetase |
| 2.4 | 1.960E-10 | 4.04E-09 | PLAC8L1 | Placenta-specific 8-like 1 |
| 2.4 | 1.100E-09 | 1.81E-08 | TRPC6 | transient receptor potential cation channel, subfamily C, member 6 |
| 2.4 | 7.130E-12 | 2.26E-10 | VLDLR | very low density lipoprotein receptor |
| 2.4 | 1.370E-08 | 1.67E-07 | ACPP | acid phosphatase, prostate |
| 2.4 | 2.630E-11 | 7.08E-10 | CHST10 | carbohydrate sulfotransferase 10 |
| 2.4 | 1.500E-13 | 7.84E-12 | DEPTOR | DEP domain containing MTOR-interacting protein |
| 2.4 | 1.760E-10 | 3.7E-09 | IGF2 | insulin-like growth factor 2 |
| 2.4 | 2.840E-11 | 7.54E-10 | MYO1C | myosin IC |
| 2.4 | 6.910E-13 | 2.95E-11 | WHSC1 | Wolf-Hirschhorn syndrome candidate 1 |
| 2.4 | 1.510E-10 | 3.26E-09 | PCDH18 | protocadherin 18 |
| 2.4 | 1.400E-09 | 2.25E-08 | PDLIM3 | PDZ and LIM domain 3 |
| 2.4 | 4.680E-06 | 2.73E-05 | EBF1 | early B-cell factor 1 |
| 2.4 | 1.300E-06 | 8.77E-06 | NID1 | nidogen 1 |
| 2.4 | 1.230E-07 | 1.13E-06 | NOX1 | NADPH oxidase 1 |
| 2.4 | 2.170E-08 | 2.47E-07 | ANXA6 | annexin A6 |
| 2.4 | 7.560E-11 | 1.76E-09 | AXIN2 | axin 2 |
| 2.4 | 1.080E-05 | 5.71E-05 | COL6A1 | collagen, type VI, alpha 1 |
| 2.4 | 4.360E-08 | 4.54E-07 | FST | follistatin |
| 2.4 | 3.160E-08 | 3.43E-07 | INCENP | inner centromere protein antigens 135/155kDa |
| 2.4 | 4.080E-09 | 5.75E-08 | KIF14 | kinesin family member 14 |
| 2.4 | 5.330E-10 | 9.64E-09 | MEIS1 | lINGrna |
| 2.4 | 6.930E-07 | 5.04E-06 | PDGFRB | platelet-derived growth factor receptor, beta polypeptide |
| 2.4 | 5.330E-08 | 5.41E-07 | DGUOK | Deoxyadenosine kinase |
| 2.4 | 9.640E-09 | 1.22E-07 | MBOAT2 | membrane bound O-acyltransferase domain containing 2 |
| 2.4 | 1.520E-08 | 1.81E-07 | SLC12A2 | solute carrier family 12 (sodium/potassium/chloride transporter), member 2 |
| 2.3 | 2.210E-09 | 3.37E-08 | PRRT1B | Proline Rich Transmembrane Protein 1B |
| 2.3 | 4.420E-13 | 2.04E-11 | TST | thiosulfate sulfurtransferase (rhodanese) |
| 2.3 | 8.820E-10 | 1.5E-08 | ACRC | acidic repeat containing |
| 2.3 | 4.780E-07 | 3.65E-06 | DAPP1 | dual adaptor of phosphotyrosine and 3-phosphoinositides |
| 2.3 | 1.100E-08 | 1.38E-07 | P2RY14 | purinergic receptor P2Y, G-protein coupled, 14 |
| 2.3 | 4.970E-09 | 6.82E-08 | PIK3C2B | phosphatidylinositol-4-phosphate 3-kinase, catalytic subunit type 2 beta |
| 2.3 | 3.080E-05 | 0.0001 | SERPINH1 | serpin peptidase inhibitor, clade H (heat shock protein 47), member 1, (collagen binding protein 1) |
| 2.3 | 1.000E-04 | 0.0004 | snoRNA RF00138 |  |
| 2.3 | 8.750E-09 | 1.12E-07 | BIRC5 | baculoviral IAP repeat containing 5 |
| 2.3 | 2.090E-10 | 4.29E-09 | CD200L | CD200 molecule-like |
| 2.3 | 2.250E-10 | 4.58E-09 | CENPI | centromere protein I |
| 2.3 | 2.880E-06 | 1.77E-05 | GPC1 | glypican 1 |
| 2.3 | 1.970E-08 | 2.27E-07 | HGF | hepatocyte growth factor (hepapoietin A; scatter factor) |
| 2.3 | 2.100E-08 | 2.4E-07 | PPDPF | pancreatic progenitor cell differentiation and proliferation factor |
| 2.3 | 1.740E-08 | 2.04E-07 | PRELP | proline/arginine-rich end leucine-rich repeat protein |
| 2.3 | 5.550E-14 | 3.46E-12 | SLC26A5 | solute carrier family 26 (anion exchanger), member 5 |
| 2.3 | 2.090E-10 | 4.29E-09 | STON2 | stonin 2 |
| 2.3 | 7.060E-10 | 1.25E-08 | CIT | citron rho-interacting serine/threonine kinase |
| 2.3 | 4.240E-07 | 3.27E-06 | GRTP1 | growth hormone regulated TBC protein 1 |
| 2.3 | 2.250E-07 | 1.89E-06 | MBOAT1 | membrane bound O-acyltransferase domain containing 1 |
| 2.3 | 8.690E-11 | 1.99E-09 | RHOC | ras homolog family member C |
| 2.3 | 9.700E-10 | 1.63E-08 | TMTC2 | transmembrane and tetratricopeptide repeat containing 2 |
| 2.3 | 3.270E-09 | 4.74E-08 | DMD | dystrophin |
| 2.3 | 4.820E-09 | 6.64E-08 | DUT | deoxyuridine triphosphatase |
| 2.3 | 1.740E-12 | 6.54E-11 | MFGE8 | milk fat globule-EGF factor 8 protein |
| 2.3 | 8.420E-07 | 5.97E-06 | SLBP | stem-loop binding protein |
| 2.3 | 7.290E-10 | 1.28E-08 | GAD1 | glutamate decarboxylase 1 (brain, 67kDa) |
| 2.3 | 3.810E-11 | 9.69E-10 | ATRNL1 | attractin-like 1 |
| 2.3 | 7.050E-07 | 5.11E-06 | CCNA2 | cyclin A2 |
| 2.3 | 8.880E-13 | 3.69E-11 | DAGLA | diacylglycerol lipase, alpha |
| 2.3 | 4.020E-05 | 0.0002 | GJA1 | gap junction protein, alpha 1, 43kDa |
| 2.3 | 8.700E-11 | 1.99E-09 | ITGAV | integrin, alpha V |
| 2.3 | 1.590E-08 | 1.89E-07 | LAMP5 | lysosomal-associated membrane protein family, member 5 |
| 2.3 | 1.940E-06 | 1.25E-05 | CAP2 | CAP, adenylate cyclase-associated protein, 2 (yeast) |
| 2.3 | 3.250E-13 | 1.55E-11 | CASP8 | caspase 8, apoptosis-related cysteine peptidase |
| 2.3 | 3.590E-11 | 9.27E-10 | FOXP4 | Forkhead Box P4 |
| 2.3 | 4.150E-07 | 3.22E-06 | GNG12 | guanine nucleotide binding protein (G protein), gamma 12 |
| 2.3 | 5.740E-08 | 5.77E-07 | PNAT3 | Arylamine N-acetyltransferase, pineal gland isozyme NAT-3 |
| 2.3 | 2.430E-09 | 3.66E-08 | SHCBP1 | SHC SH2-domain binding protein 1 |
| 2.3 | 3.830E-07 | 0.000003 | CDCA7 | cell division cycle associated 7 |
| 2.3 | 3.760E-06 | 2.24E-05 | FSTL1 | follistatin-like 1 |
| 2.3 | 2.420E-08 | 2.71E-07 | NCOA7 | nuclear receptor coactivator 7 |
| 2.3 | 3.580E-08 | 3.82E-07 | STEAP2 | STEAP family member 2, metalloreductase |
| 2.3 | 3.560E-05 | 0.0002 | EDNRB | endothelin receptor type B |
| 2.3 | 1.020E-08 | 1.28E-07 | GAS2L3 | growth arrest-specific 2 like 3 |
| 2.3 | 1.190E-07 | 1.09E-06 | GPR20 | G protein-coupled receptor 20 |
| 2.3 | 1.100E-05 | 5.83E-05 | HIST1H2B7 | Histone H2B 1/2/3/4/6 |
| 2.3 | 6.770E-07 | 4.93E-06 | JAM2 | junctional adhesion molecule 2 |
| 2.2 | 1.230E-08 | 1.51E-07 | FAM20C | family with sequence similarity 20, member C |
| 2.2 | 6.390E-09 | 8.51E-08 | KNSTRN | kinetochore-localized astrin/SPAG5 binding protein |
| 2.2 | 7.600E-11 | 1.77E-09 | PAQR8 | progestin and adipoQ receptor family member VIII |
| 2.2 | 1.080E-09 | 1.78E-08 | PDE1A | phosphodiesterase 1A, calmodulin-dependent |
| 2.2 | 5.490E-07 | 4.11E-06 | TUBB2A | tubulin, beta 2A class IIa |
| 2.2 | 1.870E-11 | 5.25E-10 | BARD1 | BRCA1 associated RING domain 1 |
| 2.2 | 6.760E-11 | 1.61E-09 | EPB41L1 | erythrocyte membrane protein band 4.1-like 1 |
| 2.2 | 3.950E-08 | 4.16E-07 | KIF11 | kinesin family member 11 |
| 2.2 | 9.930E-10 | 1.66E-08 | RHOB | ras homolog family member B |
| 2.2 | 1.090E-05 | 5.78E-05 | TAAR1 | trace amine associated receptor 1 |
| 2.2 | 8.270E-10 | 1.43E-08 | KIAA1524 | KIAA1524 |
| 2.2 | 4.830E-08 | 4.98E-07 | NT5DC2 | 5-nucleotidase domain containing 2 |
| 2.2 | 5.730E-10 | 1.03E-08 | PTTG1 | pituitary tumor-transforming 1 |
| 2.2 | 3.360E-08 | 3.61E-07 | RARRES1 | retinoic acid receptor responder (tazarotene induced) 1 |
| 2.2 | 9.590E-06 | 5.16E-05 | SLC7A5 | solute carrier family 7 (amino acid transporter light chain, L system), member 5 |
| 2.2 | 4.930E-10 | 9E-09 | AASS | aminoadipate-semialdehyde synthase |
| 2.2 | 1.570E-09 | 2.5E-08 | CCDC13 | coiled-coil domain containing 13 |
| 2.2 | 5.070E-09 | 6.93E-08 | CDX2 | caudal type homeobox 2 |
| 2.2 | 1.180E-05 | 6.18E-05 | E2F7 | E2F transcription factor 7 |
| 2.2 | 4.190E-10 | 7.84E-09 | HSP90AB1 | heat shock protein 90kDa alpha (cytosolic), class B member 1 |
| 2.2 | 2.160E-07 | 1.83E-06 | MFSD10 | major facilitator superfamily domain containing 10 |
| 2.2 | 1.810E-12 | 6.75E-11 | SLC17A9 | solute carrier family 17 (vesicular nucleotide transporter), member 9 |
| 2.2 | 3.630E-09 | 5.23E-08 | ST6GAL2 | ST6 beta-galactosamide alpha-2,6-sialyltranferase 2 |
| 2.2 | 1.460E-13 | 7.69E-12 | ATP2B4 | ATPase, Ca++ transporting, plasma membrane 4 |
| 2.2 | 1.340E-06 | 9.02E-06 | MYL9 | myosin, light chain 9, regulatory |
| 2.2 | 7.290E-14 | 4.28E-12 | PLEKHB2 | pleckstrin homology domain containing, family B (evectins) member 2 |
| 2.2 | 6.510E-07 | 4.77E-06 | SDPR | serum deprivation response |
| 2.2 | 9.480E-12 | 2.89E-10 | TERF1 | telomeric repeat binding factor (NIMA-interacting) 1 |
| 2.2 | 3.590E-10 | 6.88E-09 | TIMP4 | TIMP metallopeptidase inhibitor 4 |
| 2.2 | 2.800E-11 | 7.43E-10 | PRRX2 | paired related homeobox 2 |
| 2.2 | 2.650E-11 | 7.13E-10 | RNF144B | ring finger protein 144B |
| 2.2 | 7.910E-08 | 7.65E-07 | ROPN1L | rhophilin associated tail protein 1-like |
| 2.2 | 3.840E-09 | 5.47E-08 | SLC9A3R2 | solute carrier family 9, subfamily A (NHE3, cation proton antiporter 3), member 3 regulator 2 |
| 2.2 | 1.330E-07 | 1.21E-06 | AFF3 | AF4/FMR2 family, member 3 |
| 2.2 | 7.750E-08 | 7.53E-07 | ARVCF | armadillo repeat gene deleted in velocardiofacial syndrome |
| 2.2 | 1.210E-08 | 1.5E-07 | LHFP | lipoma HMGIC fusion partner |
| 2.2 | 2.300E-08 | 2.6E-07 | NPM3 | nucleophosmin/nucleoplasmin 3 |
| 2.2 | 3.510E-05 | 0.0002 | PDGFRA | platelet derived growth factor receptor alpha |
| 2.2 | 3.640E-11 | 9.34E-10 | TTC26 | tetratricopeptide repeat domain 26 |
| 2.2 | 1.460E-08 | 1.75E-07 | VIM | vimentin |
| 2.2 | 2.200E-10 | 4.47E-09 | BRCA1 | breast cancer 1, early onset |
| 2.2 | 3.040E-08 | 3.32E-07 | IL22RA2 | interleukin 22 receptor, alpha 2 |
| 2.2 | 9.670E-08 | 9.14E-07 | LRRC26 | leucine rich repeat containing 26 |
| 2.2 | 3.710E-07 | 2.92E-06 | NOS2 | nitric oxide synthase 2, inducible |
| 2.2 | 2.110E-06 | 1.34E-05 | PTCH2 | patched 2 |
| 2.2 | 6.740E-06 | 3.76E-05 | CYBB | cytochrome b-245, beta polypeptide |
| 2.2 | 3.280E-10 | 6.36E-09 | GRIP1 | glutamate receptor interacting protein 1 |
| 2.2 | 1.380E-09 | 2.22E-08 | MAD2L1 | MAD2 mitotic arrest deficient-like 1 (yeast) |
| 2.2 | 6.560E-07 | 4.81E-06 | NXN | nucleoredoxin |
| 2.2 | 8.690E-12 | 2.66E-10 | UNC79 | unc-79 homolog (C. elegans) |
| 2.2 | 6.410E-14 | 3.86E-12 | CAMTA1 | calmodulin binding transcription activator 1 |
| 2.2 | 4.770E-11 | 1.18E-09 | CDC45 | cell division cycle 45 |
| 2.2 | 1.260E-09 | 2.05E-08 | ENPP2 | ectonucleotide pyrophosphatase/phosphodiesterase 2 |
| 2.2 | 1.100E-07 | 1.03E-06 | FOXM1 | forkhead box protein M1 |
| 2.2 | 4.480E-10 | 8.31E-09 | GTF2H4 | general transcription factor IIH, polypeptide 4, 52kDa |
| 2.2 | 7.860E-07 | 5.62E-06 | MYBL2 | v-myb avian myeloblastosis viral oncogene homolog-like 2 |
| 2.2 | 4.090E-08 | 4.28E-07 | TWSG1 | twisted gastrulation BMP signaling modulator 1 |
| 2.1 | 2.240E-08 | 2.54E-07 | AK4 | adenylate kinase 4 |
| 2.1 | 2.090E-07 | 1.78E-06 | ANGPT1 | angiopoietin 1 |
| 2.1 | 2.620E-09 | 3.9E-08 | CENPP | centromere protein P |
| 2.1 | 5.480E-11 | 1.34E-09 | DCK | deoxycytidine kinase |
| 2.1 | 1.590E-09 | 2.52E-08 | GALNT15 | UDP-N-acetyl-alpha-D-galactosamine:polypeptide N-acetylgalactosaminyltransferase 15 |
| 2.1 | 3.590E-12 | 1.25E-10 | HIF1A | hypoxia inducible factor 1, alpha subunit (basic helix-loop-helix transcription factor) |
| 2.1 | 5.020E-08 | 5.13E-07 | KCNJ8 | potassium inwardly-rectifying channel, subfamily J, member 8 |
| 2.1 | 3.460E-11 | 8.96E-10 | LGALS3 | lectin, galactoside-binding, soluble, 3 |
| 2.1 | 7.460E-10 | 1.31E-08 | NCAPD3 | non-SMC condensin II complex, subunit D3 |
| 2.1 | 4.930E-12 | 1.65E-10 | PHEX | phosphate regulating endopeptidase homolog, X-linked |
| 2.1 | 5.690E-07 | 4.24E-06 | PPP1R17 | protein phosphatase 1, regulatory subunit 17 |
| 2.1 | 7.240E-10 | 1.27E-08 | PTTG1IP | PTTG1 interacting protein |
| 2.1 | 9.220E-06 | 4.98E-05 | RGS5 | regulator of G-protein signaling 5 |
| 2.1 | 1.890E-09 | 2.92E-08 | SLC35B2 | solute carrier family 35 (adenosine 3-phospho 5-phosphosulfate transporter), member B2 |
| 2.1 | 5.500E-14 | 3.43E-12 | SLC35C1 | solute carrier family 35 (GDP-fucose transporter), member C1 |
| 2.1 | 1.520E-08 | 1.81E-07 | SULT1C3 | sulfotransferase family, cytosolic, 1C, member 3 |
| 2.1 | 1.850E-09 | 2.87E-08 | ZDHHC2 | zinc finger, DHHC-type containing 2 |
| 2.1 | 1.080E-09 | 1.78E-08 | CAPN6 | calpain 6 |
| 2.1 | 7.390E-07 | 5.32E-06 | CKAP4 | cytoskeleton-associated protein 4 |
| 2.1 | 4.050E-08 | 4.25E-07 | ERBB4 | v-erb-b2 avian erythroblastic leukemia viral oncogene homolog 4 |
| 2.1 | 5.590E-07 | 4.17E-06 | MYLK | myosin light chain kinase |
| 2.1 | 2.290E-06 | 1.45E-05 | PRKAR2B | protein kinase, cAMP-dependent, regulatory, type II, beta |
| 2.1 | 1.640E-10 | 3.49E-09 | PYGO1 | pygopus family PHD finger 1 |
| 2.1 | 1.030E-08 | 1.29E-07 | RAD51 | RAD51 recombinase |
| 2.1 | 2.710E-07 | 2.23E-06 | RERG | RAS-like, estrogen-regulated, growth inhibitor |
| 2.1 | 9.200E-06 | 4.97E-05 | CALD1 | caldesmon 1 |
| 2.1 | 5.000E-04 | 0.0018 | FIGF | c-fos induced growth factor (vascular endothelial growth factor D) |
| 2.1 | 1.840E-09 | 2.86E-08 | KNTC1 | kinetochore associated 1 |
| 2.1 | 2.000E-04 | 0.0006 | MRC2 | mannose receptor, C type 2 |
| 2.1 | 7.330E-11 | 1.72E-09 | PPAT | phosphoribosyl pyrophosphate amidotransferase |
| 2.1 | 4.890E-13 | 2.17E-11 | TBC1D16 | TBC1 domain family, member 16 |
| 2.1 | 5.270E-09 | 7.17E-08 | YBX3 | Y-box binding protein 3 |
| 2.1 | 2.600E-11 | 7.02E-10 | BCL2L10 | Anti-apoptotic protein NR13 |
| 2.1 | 1.550E-07 | 1.38E-06 | CENPN | centromere protein N |
| 2.1 | 8.590E-06 | 4.68E-05 | FAM72A | family with sequence similarity 72, member A |
| 2.1 | 1.850E-09 | 2.87E-08 | NKD1 | naked cuticle homolog 1 (Drosophila) |
| 2.1 | 2.230E-12 | 8.07E-11 | OAZ2 | ornithine decarboxylase antizyme 2 |
| 2.1 | 8.480E-07 | 6.01E-06 | STK17A | serine/threonine kinase 17a |
| 2.1 | 2.260E-14 | 1.57E-12 | TINAGL2 | tubulointerstitial nephritis antigen like 1 |
| 2.1 | 2.730E-07 | 2.24E-06 | BEST1 | bestrophin 1 |
| 2.1 | 2.350E-10 | 4.74E-09 | CENPE | centromere protein E, 312kDa |
| 2.1 | 4.530E-08 | 4.69E-07 | GAR1 | GAR1 homolog, ribonucleoprotein |
| 2.1 | 1.900E-05 | 9.42E-05 | NRP2 | neuropilin 2 |
| 2.1 | 3.300E-09 | 4.79E-08 | UNC13C | unc-13 homolog C (C. elegans) |
| 2.1 | 2.620E-07 | 2.16E-06 | NT5C2 | 5-nucleotidase, cytosolic II |
| 2.1 | 2.440E-09 | 3.67E-08 | ACER1 | alkaline ceramidase 1 |
| 2.1 | 2.080E-06 | 1.33E-05 | CHTF8 | chromosome transmission fidelity factor 8 |
| 2.1 | 4.570E-07 | 3.5E-06 | NCAPH | condensin complex subunit 2 |
| 2.1 | 3.080E-11 | 8.1E-10 | PQLC1 | PQ loop repeat containing 1 |
| 2.1 | 1.520E-12 | 5.8E-11 | SLC51A | solute carrier family 51, alpha subunit |
| 2.1 | 1.880E-05 | 9.31E-05 | SPON1 | spondin 1, extracellular matrix protein |
| 2.1 | 3.840E-10 | 7.29E-09 | TBC1D4 | TBC1 domain family, member 4 |
| 2.1 | 2.000E-04 | 0.0006 | TNC | tenascin C |
| 2.1 | 2.420E-11 | 6.58E-10 | C7ORF50 | chromosome 14 open reading frame, human C7orf50 |
| 2.1 | 1.390E-08 | 1.69E-07 | CENPJ | centromere protein J |
| 2.1 | 7.610E-06 | 0.000042 | FOXF1 | forkhead box F1 |
| 2.1 | 3.260E-08 | 3.52E-07 | LMNB1 | lamin B1 |
| 2.1 | 4.280E-06 | 2.51E-05 | LRRC32 | leucine rich repeat containing 32 |
| 2.1 | 6.660E-11 | 1.59E-09 | BPNT1 | 3(2), 5-bisphosphate nucleotidase 1 |
| 2.1 | 3.340E-12 | 1.17E-10 | CCT8 | chaperonin containing TCP1, subunit 8 (theta) |
| 2.1 | 2.620E-09 | 3.9E-08 | DCTD | dCMP deaminase |
| 2.1 | 7.730E-09 | 1.01E-07 | MYO5C | myosin VC |
| 2.1 | 2.990E-09 | 4.39E-08 | PPA1 | pyrophosphatase (inorganic) 1 |
| 2.1 | 1.030E-11 | 3.09E-10 | PTPRK | protein tyrosine phosphatase, receptor type, K |
| 2.1 | 3.910E-08 | 4.13E-07 | S100A6 | Protein S100-A6 |
| 2.1 | 1.690E-07 | 1.49E-06 | TMEM164 | transmembrane protein 164 |
| 2.1 | 1.260E-06 | 8.54E-06 | UNC5C | unc-5 homolog C (C. elegans) |
| 2.1 | 1.710E-10 | 3.61E-09 | DEPDC1 | DEP domain containing 1 |
| 2.1 | 7.280E-10 | 1.28E-08 | FKBP9 | FK506 binding protein 9, 63 kDa |
| 2.1 | 2.070E-07 | 1.76E-06 | HSPB1 | heat shock 27kDa protein 1 |
| 2.1 | 2.000E-07 | 1.72E-06 | MCM6 | minichromosome maintenance complex component 6 |
| 2.1 | 8.860E-11 | 2.02E-09 | PKN3 | protein kinase N3 |
| 2.1 | 1.230E-06 | 8.39E-06 | PTRF | polymerase I and transcript release factor |
| 2.0 | 1.340E-08 | 1.64E-07 | ADCY9 | adenylate cyclase 9 |
| 2.0 | 1.960E-05 | 9.69E-05 | ARHGAP19 | Rho GTPase activating protein 19 |
| 2.0 | 1.220E-08 | 1.5E-07 | C4ORF19 | chromosome 4 open reading frame, human C4orf19 |
| 2.0 | 4.250E-10 | 7.94E-09 | CAV1 | caveolin-1 |
| 2.0 | 1.180E-07 | 1.09E-06 | CDC42EP3 | CDC42 effector protein (Rho GTPase binding) 3 |
| 2.0 | 5.080E-09 | 6.94E-08 | FAXC | failed axon connections homolog (Drosophila) |
| 2.0 | 6.860E-08 | 6.77E-07 | GPC2 | glypican 2 |
| 2.0 | 6.370E-10 | 1.14E-08 | HELLS | helicase, lymphoid-specific |
| 2.0 | 9.830E-10 | 1.65E-08 | IL31RA | interleukin 31 receptor A |
| 2.0 | 6.130E-06 | 3.45E-05 | NT5E | 5-nucleotidase, ecto (CD73) |
| 2.0 | 3.990E-07 | 3.11E-06 | SLC29A1 | solute carrier family 29 (equilibrative nucleoside transporter), member 1 |
| 2.0 | 8.990E-07 | 6.32E-06 | SLC37A2 | solute carrier family 37 (glucose-6-phosphate transporter), member 2 |
| 2.0 | 2.080E-11 | 5.78E-10 | ZNF516 | zinc finger protein 516 |
| 2.0 | 5.000E-08 | 5.11E-07 | CACNB2 | calcium channel, voltage-dependent, beta 2 subunit |
| 2.0 | 4.550E-08 | 4.71E-07 | ENC1 | ectodermal-neural cortex 1 (with BTB domain) |
| 2.0 | 2.340E-12 | 8.44E-11 | MGST3 | microsomal glutathione S-transferase 3 |
| 2.0 | 3.490E-11 | 9.02E-10 | MYO3AL | myosin IIIA-like |
| 2.0 | 1.920E-06 | 1.24E-05 | SOX9 | SRY (sex determining region Y)-box 9 |
| 2.0 | 1.330E-05 | 6.84E-05 | THBS1 | thrombospondin 1 |
| 2.0 | 2.300E-12 | 8.31E-11 | TKT | transketolase |
| 2.0 | 5.920E-08 | 5.91E-07 | TRIM71 | tripartite motif containing 71, E3 ubiquitin protein ligase |
| 2.0 | 1.670E-14 | 1.18E-12 | DUS1L | dihydrouridine synthase 1-like (S. cerevisiae) |
| 2.0 | 4.640E-11 | 1.16E-09 | GOT2 | glutamic-oxaloacetic transaminase 2, mitochondrial |
| 2.0 | 4.670E-10 | 8.61E-09 | HAUS1 | HAUS augmin-like complex, subunit 1 |
| 2.0 | 4.320E-08 | 4.5E-07 | HIST1H111R | histone cluster 1, H1.11R (similar to human histone cluster 1, class H1 genes) |
| 2.0 | 4.600E-05 | 0.0002 | HISTH1 | histone H1 |
| 2.0 | 2.660E-10 | 5.27E-09 | JAZF1 | JAZF zinc finger 1 |
| 2.0 | 1.820E-08 | 2.13E-07 | RHPN2 | rhophilin, Rho GTPase binding protein 2 |
| 2.0 | 1.880E-08 | 2.19E-07 | ARNT2 | aryl-hydrocarbon receptor nuclear translocator 2 |
| 2.0 | 5.550E-05 | 0.0002 | COL12A1 | collagen, type XII, alpha 1 |
| 2.0 | 1.820E-05 | 9.06E-05 | DES | DESMIN |
| 2.0 | 7.440E-11 | 1.74E-09 | HHATL | hedgehog acyltransferase-like |
| 2.0 | 3.580E-13 | 1.69E-11 | ITPR3 | inositol 1,4,5-trisphosphate receptor, type 3 |
| 2.0 | 1.080E-07 | 1.01E-06 | MAT1A | methionine adenosyltransferase I, alpha |
| 2.0 | 8.880E-11 | 2.03E-09 | GCNT1 | glucosaminyl (N-acetyl) transferase 1, core 2 |
| 2.0 | 2.040E-07 | 1.74E-06 | GOLT1B | golgi transport 1B |
| 2.0 | 1.240E-09 | 2.01E-08 | HMGA2 | high mobility group AT-hook 2 |
| 2.0 | 5.380E-08 | 5.45E-07 | IDUA | iduronidase, alpha-L- |
| 2.0 | 6.440E-07 | 4.73E-06 | KDELC2 | KDEL (Lys-Asp-Glu-Leu) containing 2 |
| 2.0 | 1.850E-06 | 0.000012 | KIF18B | kinesin family member 18B |
| 2.0 | 1.230E-07 | 1.13E-06 | MFAP5 | microfibrillar associated protein 5 |
| 2.0 | 2.690E-08 | 2.98E-07 | MTFR2 | mitochondrial fission regulator 2 |
| 2.0 | 3.130E-09 | 4.59E-08 | PTGFR | prostaglandin F receptor (FP) |
| 2.0 | 7.410E-11 | 1.73E-09 | SLC4A9 | solute carrier family 4, sodium bicarbonate cotransporter, member 9 |
| 2.0 | 1.120E-08 | 1.39E-07 | TOR4A | torsin family 4, member A |

Transcripts obtained with Transcriptomic Analysis Console (TAC) Affymetrix© software (4.0.1.36) were considered as differentially expressed transcripts (DET) when showing a >=2-fold change ratio (FCR)^1^ and a False Discovery Rate (FDR)^2^ < 0.05 between tissues.

^3^ Transcripts were annotated based on *Gallus gallus* Ensembl (release 85, [www.ensembl.org](http://www.ensembl.org)).
