# Supplementary material for: Exploring Differential Transcriptome between Jejunal and Cecal Tissue of Broiler Chickens
Source: Animals (Basel). 2019 May 7;9(5):221. doi: 10.3390/ani9050221 (PMC6562892; doi:10.3390/ani9050221)
Supplement: Supplementary file 1 [file animals-09-00221-s001.zip › supplementary files/Table S 4.docx]

**Supplementary Table 4**. Gene sets enriched in jejunal mucosa of broiler chickens, compared to cecal mucosa, ranked for the fold change ratio (FDR), q-value ≤ 0.05.

| Gene Sets of KEGG^1^-derived list | FDR q-value^2^ |
| --- | --- |
| PRIMARY_IMMUNODEFICIENCY | 0.000 |
| PEROXISOME | 0.000 |
| T_CELL_RECEPTOR_SIGNALING_PATHWAY | 0.001 |
| NATURAL_KILLER_CELL_MEDIATED_CYTOTOXICITY | 0.001 |
| PPAR_SIGNALING_PATHWAY | 0.006 |
| HEMATOPOIETIC_CELL_LINEAGE | 0.009 |
| RENIN_ANGIOTENSIN_SYSTEM | 0.010 |
| FATTY_ACID_METABOLISM | 0.010 |
| MATURITY_ONSET_DIABETES_OF_THE_YOUNG | 0.011 |
| HISTIDINE_METABOLISM | 0.020 |
| INOSITOL_PHOSPHATE_METABOLISM | 0.022 |
| PRIMARY_BILE_ACID_BIOSYNTHESIS | 0.042 |
| TRYPTOPHAN_METABOLISM | 0.042 |
| STEROID_BIOSYNTHESIS | 0.044 |

^1^The Kyoto Encyclopedia of Genes and Genomes (KEGG)

Gene set analysis was carried out on using Gene Set Enrichment Analysis (GSEA) software based on C2.CP:KEGG, C5.BP and C5.MP gene set collections (MSigDB, Broadinstitute). Normalized enriched score (NES) was calculated for each gene set. Gene sets were considered significantly enriched with False Discovery Rate (FDR)^2^ q-value ≤ 0.05 and *P*-values of NES < 0.05.
