# Supplementary material for: Exploring Differential Transcriptome between Jejunal and Cecal Tissue of Broiler Chickens
Source: Animals (Basel). 2019 May 7;9(5):221. doi: 10.3390/ani9050221 (PMC6562892; doi:10.3390/ani9050221)
Supplement: Supplementary file 1 [file animals-09-00221-s001.zip › supplementary files/Table S 5.docx]

**Supplementary Table 5**. Gene sets of KEGG^1^-derived list enriched in cecal mucosa of broiler chickens, compared to jejunal mucosa, ranked for the fold change ratio (FDR), q-value ≤ 0.05.

| Gene Sets of KEGG^1^-derived list | FDR q-value^2^ |
| --- | --- |
| CELL_CYCLE | 0.000 |
| RIBOSOME | 0.000 |
| DNA_REPLICATION | 0.000 |
| PYRIMIDINE_METABOLISM | 0.000 |
| BASAL_CELL_CARCINOMA | 0.001 |
| OXIDATIVE_PHOSPHORYLATION | 0.002 |
| ARRHYTHMOGENIC_RIGHT_VENTRICULAR_CARDIOMYOPATHY_ARVC | 0.002 |
| HEDGEHOG_SIGNALING_PATHWAY | 0.004 |
| ECM_RECEPTOR_INTERACTION | 0.004 |
| HOMOLOGOUS_RECOMBINATION | 0.006 |
| PROTEASOME | 0.008 |
| VIBRIO_CHOLERAE_INFECTION | 0.008 |
| MISMATCH_REPAIR | 0.008 |
| SPLICEOSOME | 0.009 |
| RNA_POLYMERASE | 0.012 |
| FOCAL_ADHESION | 0.014 |
| PATHOGENIC_ESCHERICHIA_COLI_INFECTION | 0.018 |
| PURINE_METABOLISM | 0.021 |
| CYSTEINE_AND_METHIONINE_METABOLISM | 0.022 |
| TASTE_TRANSDUCTION | 0.022 |
| OOCYTE_MEIOSIS | 0.028 |
| BASE_EXCISION_REPAIR | 0.040 |
| CARDIAC_MUSCLE_CONTRACTION | 0.041 |
| SYSTEMIC_LUPUS_ERYTHEMATOSUS | 0.048 |

^1^The Kyoto Encyclopedia of Genes and Genomes (KEGG)

Gene set analysis was carried out on using Gene Set Enrichment Analysis (GSEA) software based on C2.CP:KEGG, C5.BP and C5.MP gene set collections (MSigDB, Broadinstitute). Normalized enriched score (NES) was calculated for each gene set. Gene sets were considered significantly enriched with False Discovery Rate (FDR)^2^ q-value ≤ 0.05 and *P*-values of NES < 0.05.
