# Supplementary material for: Exploring Differential Transcriptome between Jejunal and Cecal Tissue of Broiler Chickens
Source: Animals (Basel). 2019 May 7;9(5):221. doi: 10.3390/ani9050221 (PMC6562892; doi:10.3390/ani9050221)
Supplement: Supplementary file 1 [file animals-09-00221-s001.zip › supplementary files/Table S 6.docx]

**Supplementary Table 6.** The first 20 gene sets of Gene Ontology - Biological Processes-derived list enriched in cecal mucosa of broiler chickens, compared to jejunal mucosa, ranked for the fold change ration (FDR), q-value ≤ 0.05.

| Gene sets of Gene Ontology - Biological Processes-derived list | FDR q-value^1^ |
| --- | --- |
| EXTRACELLULAR_MATRIX_STRUCTURAL_CONSTITUENT | 0.001 |
| NUCLEOSOME_BINDING | 0.013 |
| SNORNA_BINDING | 0.017 |
| NUCLEOSOMAL_DNA_BINDING | 0.014 |
| OXIDOREDUCTASE_ACTIVITY_ACTING_ON_A_SULFUR_GROUP_OF_DONORS | 0.012 |
| RNA_BINDING | 0.013 |
| DISULFIDE_OXIDOREDUCTASE_ACTIVITY | 0.011 |
| INSULIN_LIKE_GROWTH_FACTOR_BINDING | 0.012 |
| LAMININ_BINDING | 0.011 |
| STRUCTURE_SPECIFIC_DNA_BINDING | 0.013 |
| UNFOLDED_PROTEIN_BINDING | 0.016 |
| GROWTH_FACTOR_BINDING | 0.017 |
| PROTEIN_DISULFIDE_OXIDOREDUCTASE_ACTIVITY | 0.020 |
| CHROMATIN_DNA_BINDING | 0.024 |
| TRANSLATION_INITIATION_FACTOR_ACTIVITY | 0.028 |
| FRIZZLED_BINDING | 0.027 |
| STRUCTURAL_MOLECULE_ACTIVITY | 0.026 |
| GLYCOSAMINOGLYCAN_BINDING | 0.026 |
| STRUCTURAL_CONSTITUENT_OF_RIBOSOME | 0.027 |
| DNA_DEPENDENT_ATPASE_ACTIVITY | 0.033 |

Gene set analysis was carried out on using Gene Set Enrichment Analysis (GSEA) software (MSigDB, Broadinstitute). Normalized enriched score (NES) was calculated for each gene set. ^1 1 1^Gene sets were considered significantly enriched with False Discovery Rate (FDR) q-value ≤ 0.05 and *P*-values of NES < 0.05.
