# Supplementary material for: Exploring Differential Transcriptome between Jejunal and Cecal Tissue of Broiler Chickens
Source: Animals (Basel). 2019 May 7;9(5):221. doi: 10.3390/ani9050221 (PMC6562892; doi:10.3390/ani9050221)
Supplement: Supplementary file 1 [file animals-09-00221-s001.zip › supplementary files/Table S 7.docx]

**Supplementary Table 7**. Non-coding differentially expressed transcripts in jejunal or cecal mucosa of broiler chickens, with a log2-fold change ratio (FCR) ≥2.

| Cecum  Avg (log2)^1^ | Jejunum Avg (log2)^1^ | Fold Change^2^ | Name |
| --- | --- | --- | --- |
| Jejunum |  |  |  |
| 4.56 | 6.91 | -5.1 | snoRNA RF00004 |
| 3.9 | 5.21 | -2.48 | gga-mir-1783 |
| 2.53 | 3.82 | -2.45 | gga-mir-1654-1 |
| 5.88 | 6.96 | -2.11 | snoRNA RF00431 |
| 3.58 | 4.64 | -2.08 | gga-mir-1416 |
| 2.15 | 3.16 | -2.02 | gga-mir-215 |
| Cecum |  |  |  |
| 7.71 | 4.16 | 11.7 | gga-mir-196-4 |
| 4.09 | 2.14 | 3.87 | gga-mir-196-1 |
| 6.88 | 5.49 | 2.61 | gga-mir-1732-1 |
| 7.8 | 6.58 | 2.33 | snoRNA RF00138 |
| 5.05 | 4.02 | 2.05 | gga-mir-1800 |

^1^ Average value of log2 FCR of non-coding differentially expressed transcripts (DET) in jejunum and cecum

^2^ Non-coding transcripts obtained with Transcriptomic Analysis Console (TAC) Affymetrix© software (4.0.1.36) were considered as DET between tissues (jejunum and cecum) with a log2-fold change ratio (FCR) ≥2
